# Expression Partitioning of Duplicate Genes at Single Cell Resolution in *Arabidopsis* Roots

**DOI:** 10.3389/fgene.2020.596150

**Published:** 2020-11-03

**Authors:** Jeremy E. Coate, Andrew D. Farmer, John W. Schiefelbein, Jeff J. Doyle

**Affiliations:** ^1^Department of Biology, Reed College, Portland, OR, United States; ^2^National Center for Genome Resources, Santa Fe, NM, United States; ^3^Department of Molecular, Cellular, and Developmental Biology, University of Michigan, Ann Arbor, MI, United States; ^4^School of Integrative Plant Science, Plant Biology Section, Cornell University, Ithaca, NY, United States

**Keywords:** gene duplication, single cell RNA-seq, cell type, cell state, polyploidy, expression subfunctionalization

## Abstract

Gene duplication is a key evolutionary phenomenon, prevalent in all organisms but particularly so in plants, where whole genome duplication (WGD; polyploidy) is a major force in genome evolution. Much effort has been expended in attempting to understand the evolution of duplicate genes, addressing such questions as why some paralog pairs rapidly return to single copy status whereas, in other pairs, both paralogs are retained and may diverge in expression pattern or function. The effect of a gene – its site of expression and thus the initial locus of its function – occurs at the level of a cell comprising a single cell type at a given state of the cell’s development. Using *Arabidopsis thaliana* single cell transcriptomic data we categorized patterns of expression for 11,470 duplicate gene pairs across 36 cell clusters comprising nine cell types and their developmental states. Among these 11,470 pairs, 10,187 (88.8%) had at least one copy expressed in at least one of the 36 cell clusters. Pairs produced by WGD more often had both paralogs expressed in root cells than did pairs produced by small scale duplications. Three quarters of gene pairs expressed in the 36 cell clusters (7,608/10,187) showed extreme expression bias in at least one cluster, including 352 cases of reciprocal bias, a pattern consistent with expression subfunctionalization. More than twice as many pairs showed reciprocal expression bias between cell states than between cell types or between roots and leaves. A group of 33 gene pairs with reciprocal expression bias showed evidence of concerted divergence of gene networks in stele vs. epidermis. Pairs with both paralogs expressed without bias were less likely to have paralogs with divergent mutant phenotypes; such bias-free pairs showed evidence of preservation by maintenance of dosage balance. Overall, we found considerable evidence of shifts in gene expression following duplication, including in >80% of pairs encoding 7,653 genes expressed ubiquitously in all root cell types and states for which we inferred the polarity of change.

## Introduction

According to Lynch and Trickovic (2020, p. 1861), “One of the last uncharted territories in evolutionary biology concerns the link with cell biology. Because all phenotypes ultimately derive from events at the cellular level, this connection is essential to building a mechanism-based theory of evolution.” As a candidate for building such a connection to cell biology, it would be difficult to identify a more important molecular evolutionary process than gene duplication, whose key role has been universally recognized since the classic paper of Ohno (1970), half a century ago. Gene duplication occurs at high frequency, estimated at 0.01 duplications per gene per million years in eukaryotic genomes, and large numbers of recently formed paralogs are found in typical animal, fungal, and plant genomes (Lynch and Conery, 2000; Lynch et al., 2001). Among eukaryotes, plant genomes, in particular, are characterized by massive levels of duplication, thanks to waves of whole genome duplication (WGD, polyploidy). Recent estimates place the number of known plant WGD events at the genus level or above at over 250, and most plant lineages have experienced multiple cycles of polyploidy (Van de Peer et al., 2017; Leebens-Mack et al., 2019). Because they are the products of both small scale duplications (SSD) and WGD, plant gene families can be very large and complex (Panchy et al., 2016).

The fate of most paralogs, whether produced by SSD or WGD, is pseudogenization and eventual loss, through mutations that inactivate redundant copies during the “fixation phase” of a duplicate gene’s life cycle (Innan and Kondrashov, 2010; Xie et al., 2019). Various mechanisms have been hypothesized that can preserve paralog pairs by making both copies of the gene indispensable (Innan and Kondrashov, 2010; Panchy et al., 2016; Qiao et al., 2019). These mechanisms can differ for SSD vs. WGD, even operating in different directions in the case of dosage effects (Papp et al., 2003; Freeling, 2009; Birchler and Veitia, 2010, 2012, 2014). Understanding why and how gene pairs are retained is complicated in part because in many cases competing hypotheses are difficult to distinguish from one another in terms of their predictions (Innan and Kondrashov, 2010). Obtaining empirical data for testing these hypotheses is not easy. Several of the models involve “function,” a term that can be difficult to define.

**FIGURE 1:**
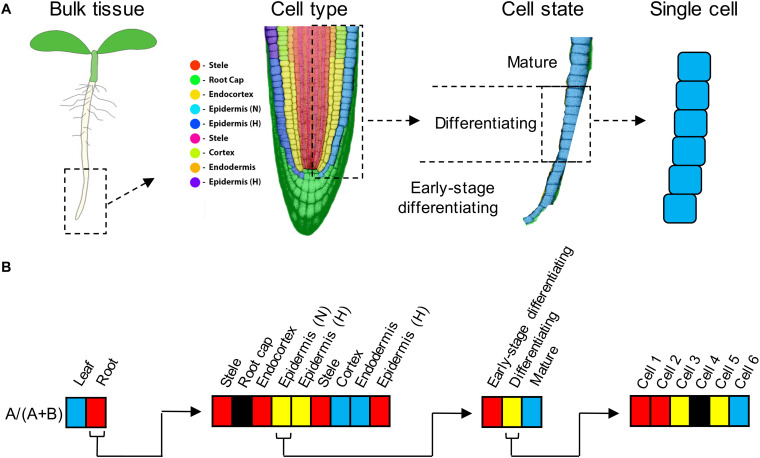
**(A)** Expression subfunctionalization is the partitioning of ancestral expression profiles between paralogs. Partitioning can occur at the level of tissue types (e.g., leaves vs. roots), but also at increasingly finer scales, including at the level of cell type (in roots, roughly corresponding to the nine cell clusters identified by Ryu et al., 2019), or cell state (e.g., developmental gradients within a single cell type such as non-hair epidermal cells), and even among single cells. Under cell type, “Epidermis (N)” refers to non-hair (atrichoblast) epidermal cells, and “Epidermis (H)” refers to root hair (trichoblast) epidermal cells (Ryu et al., 2019). There are two clusters labeled “Epidermis (H)” because the available marker genes assigned them both to the same cell type, but their transcriptional profiles were sufficiently distinct (most likely due to differences in developmental stage) that the clustering algorithm separated them. There are two clusters labeled “Stele” for similar reasons. **(B)** Examples of how expression partitioning between paralogs (designated “A” and “B”) can occur at different levels. Expression bias is indicated by shading (red = expression biased toward paralog A [specifically, A/(A + B) > 0.9], blue = expression biased toward paralog B [A/(A + B) < 0.1], yellow = unbiased expression, black = neither paralog expressed). Left, in this example, paralog A expression predominates in roots, whereas paralog B expression predominates in leaves. Middle left, within root tissue, paralogs A and B exhibit partitioning by cell type. Middle right, within the non-hair epidermal cell type, paralogs A and B exhibit partitioning by cell state (i.e., developmental stage). Right, within the “differentiating” stage of non-hair epidermal cell type, A and B paralog expression is partitioned among individual cells (paralog A predominating in cells 1–2, and paralog B expression predominating in cell 6).

Gene expression is often used as a proxy for gene function when assessing the fates of duplicate genes (e.g., Panchy et al., 2019), with biased expression of paralogs of a duplicated gene providing evidence for sub- or neofunctionalization. Expression proportional to gene copy number is a key component of models that involve preservation of duplicates via stoichiometric constraints (Coate et al., 2016; Song et al., 2020). Expression occurs in the nuclei of individual cells, which comprise different “cell types,” the existence of which is taken as a given in the molecular and developmental biology literature, but which are difficult to define. Although there has been recent theoretical progress in how cell types originate and evolve (e.g., Arendt et al., 2016; Liang et al., 2018; Yuan et al., 2020), Vickaryous and Hall (2006) liken the problem of defining “cell type” to defining “species” – an endless source of controversy in evolutionary biology. One major complication is that although cell identity may be stable, “the same cell type can exhibit a range of different phenotypes (states)” (Morris, 2019) in response to diverse physiological or developmental stimuli. Each state of each cell type has its own characteristic transcriptome, so it is at the level of individual cell types and states that we should find the most precise transcriptomic data to explore the expression-based mechanisms that preserve duplicate genes. Studies at the tissue or organ level aggregate different cell types, obscuring patterns of gene expression that may be of interest (Figure 1; Efroni and Birnbaum, 2016; Libault et al., 2017).

Until recently, plant studies at the single cell type level were mostly limited to isolation of individual cell types by flow sorting (e.g., Birnbaum et al., 2003), or to cell types for which large populations of pure cells can be obtained, such as root hairs (e.g., Qiao and Libault, 2013; Hossain et al., 2015) and cotton seed fibers (e.g., Shi et al., 2006; Gou et al., 2007; Taliercio and Boykin, 2007). Cotton fibers have been studied extensively in the context of polyploidy (e.g., Hovav et al., 2008a; Yoo and Wendel, 2014; Gallagher et al., 2020). However, bulked samples of a single cell type, even when collected at different developmental stages, may still miss some details of transitions among cell states. Additionally, by focusing on a single cell type, such studies are blind to any expression partitioning between paralogs that might have occurred across cell types.

Single cell methods that have revolutionized biology continue to develop and promise ever more powerful and precise data (Lähnemann et al., 2020). In plants, several groups recently published single cell transcriptomic studies of *Arabidopsis* roots (Denyer et al., 2019; Jean-Baptiste et al., 2019; Ryu et al., 2019; Shulse et al., 2019; Zhang et al., 2019) that not only identified known cell types, including cell types represented by small numbers of cells that would be missed in conventional transcriptomic studies, but also revealed cells with distinctive transcriptomes not readily assigned to known cell types, and subdivided cell types into different developmental states. These data provide a potential resource for exploring gene duplication events at the single cell level in *A. thaliana*. Much is known about gene duplication in this model species, including classification of the origins of its thousands of paralogous gene pairs by various mechanisms of single gene duplication (SSD) and whole genome duplication (WGD, polyploidy) (e.g., Wang et al., 2013; Hao et al., 2018; Qiao et al., 2019) and the degree to which paralogs from many pairs have diverged functionally (Hanada et al., 2009; Panchy et al., 2019). Much is also understood about the process of biased genome fractionation following WGD 30–40 million years ago (MYA), which has led to the retention of only a subset of duplicated genes, unequally distributed across the homoeologous subgenomes of *A. thaliana* (e.g., Cheng et al., 2018; Emery et al., 2018; Liang and Schnable, 2018).

The availability of *Arabidopsis* root single cell data allowed us to explore expression patterns of over 11,000 paralogous gene pairs at a finer scale than has previously been reported. We find many examples of expression differentiation of paralogous genes at the level of cell types and states within a single organ, similar to what Adams et al. (2003) found at the level of whole organs comprising the flower. A large fraction of gene pairs show evidence of evolutionary shifts in expression between paralogs, including over 75% of the over 1,500 gene pairs with one or both paralogs ubiquitously expressed in all root cell types and states. Patterns of expression of gene pairs from whole genome duplications vs. single gene duplications are mostly consistent with expectations, with evolutionary differentiation of expression between paralogs more common following single gene duplications than following polyploidy, and pairs from WGD events showing evidence of preservation by dosage balance (e.g., Freeling, 2009; Panchy et al., 2016; Tasdighian et al., 2017; Defoort et al., 2019; Qiao et al., 2019). We find evidence of concerted divergence of gene networks between different root cell types. We also show that different cell types have responded differently to gene and genome duplications in the degree to which they deploy one or both paralogs in their transcriptomes.

## Materials and Methods

### Single Cell Datasets

Illumina sequence data for NCBI SRA experiments SRX5074330-SRX5074332 corresponding to scRNA-seq data from three wild-type replicates of *Arabidopsis* roots from Ryu et al. (2019) were processed with the 10× Genomics Cell Ranger v3.1.0 count pipeline, run independently on the data for each replicate to produce unique molecular identifier (UMI) raw counts matrices against the TAIR10 genome using Araport11 annotations (Cheng et al., 2017). Custom scripts (available from^1^) were used to produce per-cluster UMI counts for each gene, summing the contributions from all cells assigned to the 9 “superclusters” presented in Ryu et al. (2019) and separately for 36 root cell clusters (“RCCs”) derived from those 9 initial superclusters using the Seurat software package (Butler et al., 2018; FindClusters function) with default parameters (perplexity = 30, random seed = 1) and a resolution of 3.5. These per-supercluster and per-RCC gene UMI count matrices formed the basis of subsequent analysis of expression bias between duplicated gene pairs. Specific cell types and differentiation states were assigned to each of the 36 RCCs from the 9 initial superclusters (Supplementary Table 1) using previously defined marker genes (Ryu et al., 2019).

To filter out spurious expression signals (resulting, for example, from doublets or from cell-free RNA), we required at least one UMI from two or more cells in a given cluster for a gene to be considered expressed in the context represented by the cluster. In some cases, we also analyzed the data using the minimally restrictive expression threshold of ≥1 UMI from ≥1 cell to assess how strongly additional filtering affects the results.

The cells in the Ryu et al. (2019) study from which the data were taken were derived from protoplasted root tissues, and would thus be subject to some level of protoplasting-induced changes in gene expression relative to untreated tissues (Birnbaum et al., 2003). We considered that altered responses to the protoplasting treatment were within the scope of what could be considered paralog expression divergence, and we retained duplicate pairs involving such genes in the subsequent analyses.

### Sequence Read Archive (SRA) Datasets

A total of 214 RNA-seq datasets for *Arabidopsis thaliana* used in Panchy et al. (2019) were obtained from NCBI SRA (Leinonen et al., 2011) and aligned to the TAIR 10 genome using hisat2 v2.1.0 (Kim et al., 2019) against an index built with splice sites and exons derived from Araport11 annotations. TPM values derived by stringtie v2.0.6 (Kovaka et al., 2019) were then converted to raw counts for the Araport 11 genes (Supplementary Table 2). These data were used to assess if genes not expressed in the Ryu et al. (2019) scRNA-seq data were expressed in other tissues and/or conditions. Of the 214 SRA libraries, 37 were generated from leaf tissue only (832 million reads total) and 31 were from root tissue only (290 million reads total). Counts were summed across all samples in each class (leaf vs. root) to provide high-coverage bulked data sets to compare paralog expression at the level of contrasting tissue types, in the same manner as for the clustered single-cell data, as described below.

### Biased Expression of Paralogs

For gene pairs identified by Wang et al. (2013), we determined whether the paralogs showed biased expression (analogous to “biased homoeolog expression” of Grover et al., 2012) in the context of each cluster, using a UMI cutoff of 9:1. We chose 9:1 as a stringent threshold for biased expression because our primary interest was in identifying cases of extreme imbalance in paralog expression, consistent with expression subfunctionalization. The two paralogs of the gene pair were then designated “single-cell A” (scA) and “single-cell B” (scB), with scA being the dominant paralog showing higher expression in the greatest number of clusters.

In order to exclude from consideration gene pairs whose apparent bias may be insignificant relative to random sampling deviations, we considered the counts characterizing each gene pair as representing the outcome of a Bernoulli trial, where the abundance of reads relative to its partner determines the probability of each outcome (i.e., sampling a particular partner when a read is chosen from one of the pair). In order to determine confidence in the estimate of the probability given a specific number of trials (i.e., the summed read count for the pair), the Wilson (1927) score interval estimation was used to provide a 95% confidence interval around the expression bias value estimated from the read counts. Considered from the perspective of the dominant gene in a putatively biased pair, the lower bound on the confidence interval can be used as the minimum level of bias at the chosen confidence level, and if this minimum level of bias falls below the 9:1 threshold ratio for considering a gene pair to exhibit bias, it was removed from consideration when calculating the fixation and balance indices described below.

For each paralog pair we calculated two indices to describe its pattern of expression across cell clusters:

• The Expression Fixation index (F_ex_) measures the degree of bias in the expression of paralogs of a given pair across the cell clusters. F_ex_ = N_fix_/(number of cell clusters expressing at least one paralog above a cutoff threshold), where N_fix_ is the number of clusters for which one paralog (either one) is “fixed” (is preferentially expressed at or above the 9:1 threshold).
• The Balance Index (B_fix_) is calculated for any paralog pair for which at least one cluster is fixed for a paralog, and measures the degree to which one paralog dominates the expression across the clusters. B_fix_ = 2^∗^(number of clusters fixed for scB)/(number of clusters fixed for either paralog). B_fix_ runs from 0–1; cases with no fixation of either paralog (paralog pairs with F_ex_ = 0) have no B_fix_ score (N/A).

**FIGURE 2:**
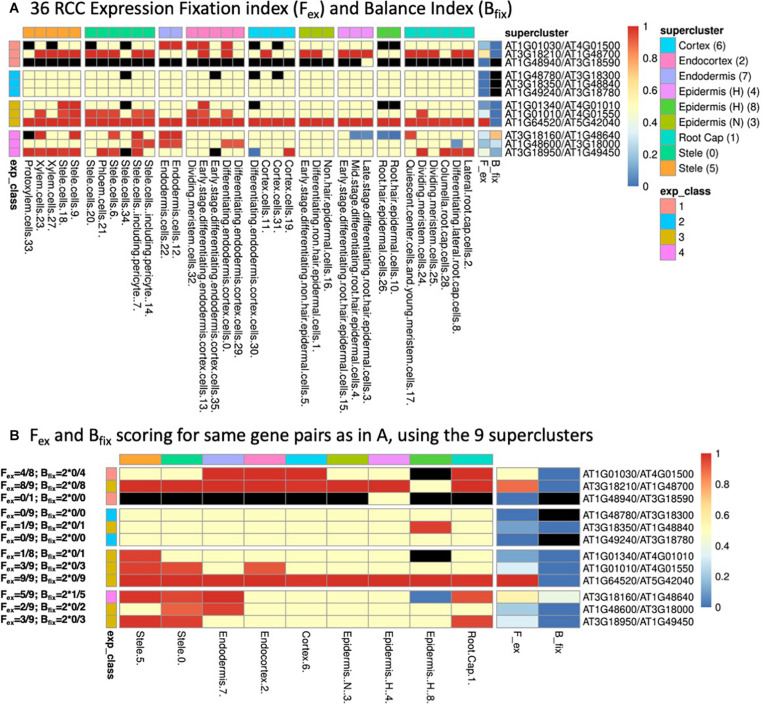
**(A)** Heatmap of 36 root cell clusters (RCCs) from nine Ryu et al. (2019) superclusters, showing examples of selected gene pairs illustrating F_ex_ and B_fix_ (last two columns before gene pair designations) and the four expression classes (defined in Table 1). Cell colors: for RCCs, red cells have dominant paralog scA fixed (expressed above the 9:1 threshold at 95% confidence level, or, in the case of Class 1, expressed at 1:0); blue cells have paralog scB fixed; yellow cells have at least one of the paralogs expressed, but do not meet the statistical criterion for biased expression; black cells have no expression of either paralog. For F_ex_ and B_fix_ the cell values for each index run from 0–1 and are colored continuously from blue to red as indicated by the scale at the right of the figure. RCCs are grouped according to the superclusters from which they were derived (as indicated by column heading colors), while gene pairs are grouped according to their expression classes (as indicated by row heading colors). **(B)** Corresponding heatmap of the same gene pairs as shown in **(A)** using the nine Ryu et al. (2019) superclusters as the basis for assessment of expression bias. Expression class colors are as in **(A)**. Examples of the calculation of F_ex_ and B_fix_ values for each gene pair based on the expression-biased contexts detected in each case are given to the left of the figure.

Examples are given in Figure 2, with explicit calculations of the indices given for the gene pairs in panel B and the continuous color scale at the right indicating the numerical values (black indicates an undefined value due to a zero appearing in the denominator). As may be seen by comparing panels A and B of Figure 2, which represent several gene pairs characterized at both the 36 RCC level (Figure 2A) and the 9 supercluster level (Figure 2B), the values of the indices can differ relative to the granularity of the clustering at which a given gene pair is considered. In general, the finer resolution of the 36 RCC allows more sensitivity in observing biased contexts that would otherwise be obscured by aggregation with other less-biased or oppositely-biased contexts (e.g., the last row in each table representing gene pair AT3G18950/AT1G49450). In contrast, in some cases going to a finer level of clustering can actually diminish the statistical power of detecting bias due to the lower UMI counts associated with each of the individual RCCs (e.g., the fifth row in each table representing gene pair AT3G18350/AT1G48840).

### Classification of Paralogs by Expression Pattern

We assigned paralog pairs to one of five classes based on their expression patterns across the 36 RCCs (Table 1 and Figure 2A). Genes were also assigned to equivalent classes at the nine cluster level (e.g., Class 1 if only one of the two copies was expressed at the 9-supercluster level; Figure 2B). As noted in Table 1, these classes depend not only on our previously stated definition of expression/non-expression of each gene (UMI from two or more cells) but also on the values of the F_ex_ and B_fix_ indices, which characterize patterns of *statistically significant* bias. Thus, for example, in Figure 2A, for the Class 1 gene pairs which by definition involve expression of only the scA paralog, we see a mixture of contexts in which some are black (implying no expression of either paralog), some are red (implying biased expression meeting the Wilson criterion) and some are yellow (implying that expression did not meet the statistical test for bias, despite the fact that, in the context of Class 1, only scA met the definition of expression). Again, a comparison of Figure 2 panels A and B shows that the expression class assigned to a given gene pair can change when the level of resolution of the clusters is altered.

**TABLE 1:** Expression classes of duplicate gene pairs.

Class	Copies expressed	F_ex	B_fix	Summary
0	0	0	0	No expression in any RCC
1	1	≥0*	0	Only 1 copy expressed
2	2	0	0	Both copies expressed, no fixation
3	2	>0	0	Both copies expressed, some fixation, no balance
4	2	>0	>0	Both copies expressed, balanced fixation (subfunctionalization?)

*In some Class 1 cases, despite only one copy being expressed, no clusters have significant fixation due to low read counts.

### Fixation Similarities and Differences Across Clusters

Heatmaps were produced on the results of calculating biased expression for each gene pair across all clusters, by using the R package “Pretty Heatmaps” (pheatmap v1.0.12). The annotations option of pheatmap was used to denote the expression class (Table 1).

### Single Cell Measurements of Paralog Usage

In order to look at possible bias between duplicated genes at the level of single cells, we again applied the Wilson (1927) test on the UMI counts for the duplicated genes at the level of individual cells. In this case, due to the low counts obtained and in order not to confute subsequent tests we extended the states recorded by the test to distinguish not only between biased and unbiased but also cases where the counts were too low to determine whether a particular gene pair fell in one class or another in a given cell. Cells in the latter case were excluded from subsequent analyses. For the remainder, gene pairs could be tallied with respect to the number of cells in a given cluster that showed bias for one or the other of the genes, were unbiased with respect to the expression of the genes, or showed no expression of either gene (i.e., had a UMI count of zero for both). The number of cells falling into each category were then used to test for significant overlap of the lists of cells expressing each of the two genes, using Fisher’s exact test in the manner of the GeneOverlap Bioconductor package (Shen and Sinai, 2019), while additionally testing for significant non-overlap (i.e., a tendency for cells expressing one paralog to not express the other paralog) by utilizing the alternate tail of the hypergeometric distribution.

### K_a_/K_s_ Value Determination for Paralogs

In order to assess whether different classes of paralogs based on patterns of expression showed significant differences in terms of protein coding divergence, we used the implementation of the Nei-Gojobori algorithm (Nei and Gojobori, 1986) provided by the Bio:Align:DNAStatistics module of BioPerl (Stajich et al., 2002) through wrapper scripts available in the MCScanX software distribution (Wang et al., 2012) but run across all the *Arabidopsis* gene duplicate pairs classified by Wang et al. (2013) including non-syntenic SSD pairs.

### Gene Ontology Enrichment Analysis

GO term enrichment was assessed using ThaleMine’s GO enrichment analysis widget with default parameters. Specifically, GO term representation in specific gene sets was compared to representation in the full *Arabidopsis* gene set (Araport 11; Cheng et al., 2017), and tested for significance at *p* < 0.05 (Fisher’s Exact Test with Holm-Bonferroni correction).

## Results

### *Arabidopsis* Root Cell Clusters Each Express Over 35% of Genes in the Genome

**FIGURE 3:**
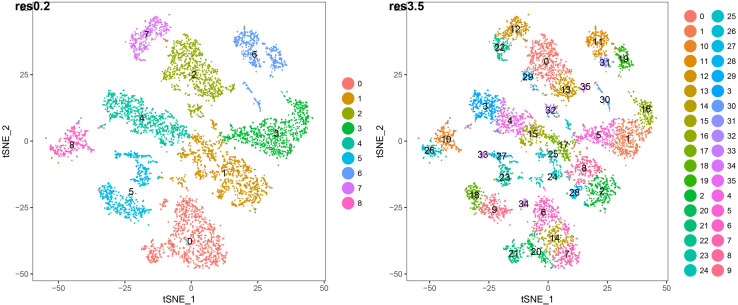
t-SNE plots of 7522 wild-type *Arabidopsis* root single-cell transcriptomes clustered at two different resolutions. The lower resolution generates 9 major clusters (“superclusters”; roughly representing “cell types”) and the higher resolution generates 36 root cell clusters (RCCs; roughly representing “cell states”). The specific cell type/state assignments for each cluster are provided in Figure 2. Additional details are provided in Supplementary Table 1.

We studied both the 9 root cell supercluster transcriptome data of Ryu et al. (2019) and transcriptomes of the 36 root cell clusters (RCCs) derived from those superclusters. These two datasets are strongly nested, with the 9 superclusters broadly corresponding to cell types and the 36 RCC data including developmental states of those types (Figure 3 and Table 1). We primarily report results from the 36 RCCs here (9 supercluster counts data and 36 RCC counts data are available in Supplementary Table 3).

We explored two different thresholds for determining whether a gene was expressed in an RCC: at least 1 UMI per cluster vs. the more stringent cutoff of expression in two or more cells of a given RCC. These thresholds were chosen to accommodate different technical issues with single-cell RNA-seq droplet-based methods (Luecken and Theis, 2019). On the one hand, technical dropout (Bhargava et al., 2014) leads to reduced capture for low abundance transcripts, suggesting a relaxed threshold for counts relative to bulk RNA-seq; conversely, the possibility of capturing multiple cells of differing types in a single droplet (“doublets”) suggests a need to guard against false positives generated by this phenomenon. We found that counts derived from these two thresholds were strongly correlated (*R*^2^ = 0.997) but 7–18% lower for the two cell cutoff than for the 1 UMI cutoff (Supplementary Figure 1); we report numbers using the more stringent cutoff throughout.

**FIGURE 4:**
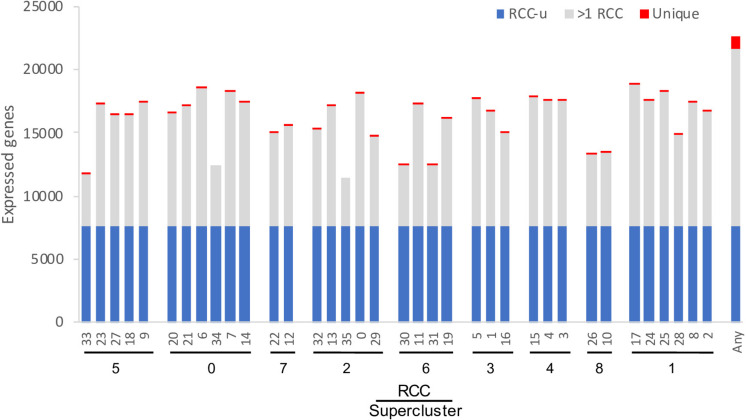
Number of genes expressed per cluster. The 36 RCCs are grouped by Ryu et al. (2019) supercluster (roughly representing cell types and developmental states; Figure 1), and ordered roughly from the interior (stele) to the exterior (epidermis) and tip (root cap) of the root. RCC-ubiquitous (RCC-u) is the subset of genes expressed in all 36 RCCs, “>1 RCC” is the subset expressed in 2–35 RCCs, and “Unique” is the subset expressed in only one RCC. “Any” is the union of all genes expressed in at least one of the 36 clusters.

After excluding loci annotated as “novel transcribed regions,” “pseudogenes,” “transposable element genes,” and organelle-encoded genes, there are 32,548 genes in the most recent annotation of the *Arabidopsis* genome (Araport11: Cheng et al., 2017). Of these, 22,669 (70%) were expressed in at least one of the 36 RCCs, with each RCC expressing 35–58% of these genes (Figure 4). These percentages are comparable to *Arabidopsis* pollen cell stages (32–51% of microarray features; Honys and Twell, 2004), and are somewhat lower than those for cotton fiber cells, which transcribe from 75–94% of the genome’s genes depending on developmental stage (Hovav et al., 2008b). Differences in the number of genes expressed per cell cluster were in many cases statistically significant, but these differences were largely driven by differences in cell count per RCC (Supplementary Figure 2). Approximately 35% of root-expressed genes (∼22% of all *Arabidopsis* genes) were expressed in all 36 RCCs (“RCC-ubiquitous,” subsequently referred to as “RCC-u”); 1,059 genes (4.7% of root-expressed genes) were uniquely expressed in only one RCC (Figure 4).

### Many Gene Pairs Show Biased Paralog Expression in Root Cell Clusters, and Different Duplication Types Show Different Expression Patterns

Wang et al. (2013) identified 11,630 gene pairs in the *Arabidopsis* genome, and classified them as being duplicated either by WGD or by SSD (Supplementary Table 3). They further subdivided the WGD class into products of the alpha (most recent, around 31.8–42.8 MYA; Edger et al., 2018), beta (85–92.2 MYA; Edger et al., 2018), and gamma events (115–120 MYA; Jiao et al., 2012), and the SSD class into tandem duplicates, proximal duplicates, and two subclasses of transposed duplicates (younger than 16 MYA vs. older). 160 of the Wang et al. (2013) pairs contain obsolete gene models and were excluded from subsequent analyses. We divided the remaining 11,470 paralog pairs into five RCC expression classes according to whether neither paralog (Class 0), only one paralog (Class 1), or both paralogs (Classes 2, 3, and 4) were expressed in at least one cluster; Classes 2, 3, and 4 were distinguished from one another by patterns of biased paralog expression estimated at a 9:1 ratio (Figure 2 and Supplementary Table 3).

**FIGURE 5:**
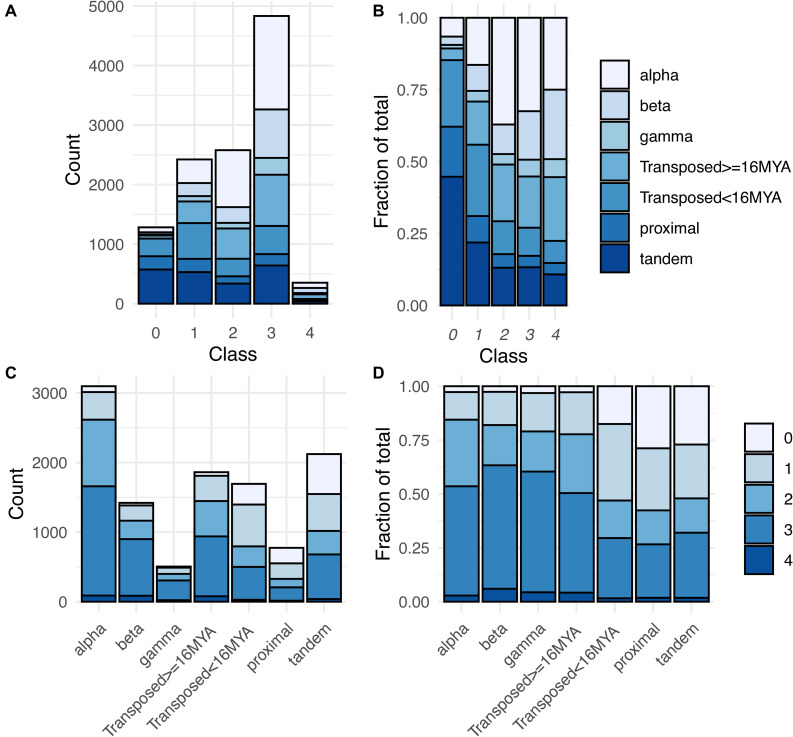
Distribution of expression classes and duplication types for gene pairs. **(A)** Counts of gene pairs by expression class, broken down by duplication mechanism. **(B)** Fraction of gene pairs in each expression class produced by each duplication mechanism. **(C)** Counts of gene pairs by duplication mechanism, broken down by expression class. **(D)** Fraction of gene pairs from each duplication mechanism assigned to each expression class.

Overall, 10,187 of these gene pairs (88.8% of total pairs) belonged to Classes 1–4, having one or both paralogs expressed in at least one RCC (Supplementary Table 4). This percentage is similar to but significantly lower than the expectation for drawing at least one member of a gene pair from the 70% of *Arabidopsis* genes expressed in root cell clusters (90.8%; *χ*^2^ = 24.3, *p* < 0.001). Notably, however, 67.7% (7,764 pairs) had both copies expressed in root cell clusters, significantly higher than the random expectation of 48.5% (*χ*^2^ = 867.4, *p* < 0.001). There was a clear distinction between WGD and local SSD (proximal and tandem) pairs with regard to these percentages. WGD pairs were significantly more likely to express at least one copy (≥96.8%; *χ*^2^ ≥ 16.23, *p* < 0.001) and to express both copies (≥79.0%; *χ*^2^ ≥ 101.6, *p* < 0.001) than expected by chance, whereas local SSD pairs were significantly less likely to express at least one copy (≤72.9%; *χ*^2^ ≥ 97.0, *p* < 0.001) and less than or similarly likely to express both copies (proximal: 42.4%; *χ*^2^ = 5.76, *p* = 0.016; tandem: 47.9%; *χ*^2^ = 0.136, *p* = 0.712). Older duplicates created by transposable elements (TEs) exhibited a similar pattern to WGD duplicates and younger TEs exhibited a similar pattern to local SSD duplicates (Figure 5 and Supplementary Table 4).

The expression breadth of pairs (number of clusters in which a pair is expressed) also differed by duplication type (Supplementary Figure 3). For all three WGD types, around 44–48% of paralog pairs had one or both paralogs expressed in all 36 RCCs (“RCC-u pairs”), and 30–38% of genes were RCC-u. Only 4–6% of pairs had no expression of either paralog in any RCC (Class 0 pairs; Table 1, Figure 5, and Supplementary Figure 3), comprising 8–12% of all genes. In contrast, tandem and proximal SSD types had no expression of 25–30% of pairs and around 35–40% of their individual genes in any RCC and fewer than 20% of pairs and 12% of their genes were expressed in all 36 RCCs. Transposed duplicates were intermediate between these two groups, with older pairs again behaving more like WGD types (44% of pairs and 30% of individual genes expressed in all RCCs; 2% of pairs and 12% of genes not expressed in any RCC) and younger transposed pairs behaving more like the other SSD types (28% of all pairs and 17% of genes expressed in all 36 RCCs; 18% of pairs and 33% of genes not expressed in any RCC).

Class 1 gene pairs have the same paralog exclusively expressed in all root cell clusters in which either paralog is expressed (balance index [B_fix_] = 0) (Figure 2 and Table 1). The same dichotomy between duplication types observed for Class 0 gene pairs was also observed for Class 1: Only around 10–20% of WGD and older transposed duplicate pairs belonged to this class, vs. 25–35% of tandem, proximal, and younger transposed SSD classes (Figure 5 and Supplementary Table 4).

In a Class 1 pair, only one paralog (by definition the dominant paralog, scA) is expressed in roots. ScB shows no expression in any RCC, and could either be a pseudogene, or could be a functional gene expressed in other contexts than the roots studied here. We thus looked at expression of Class 1 scB paralogs in 214 RNA-seq experiments obtained from the SRA database (Leinonen et al., 2011; Supplementary Table 2). We found that in 93% of Class 1 gene pairs, the scB paralog was expressed in at least one SRA dataset, and 91% were expressed in non-root SRA libraries (requiring at least 5 reads to be considered expressed) (Supplementary Table 5). These results indicate that the majority of Class 1 genes (90–93%) represent cases where expression has been partitioned between paralogs since their divergence, with scA being the only copy now expressed in roots grown under the conditions used by Ryu et al. (2019). Although this suggests that many of these scB paralogs could be functional, many could still be pseudogenes, because around a third of plant pseudogenes are expressed (Xie et al., 2019). Lloyd et al. (2018) found that expression level and, particularly, expression breadth across tissues, were the best predictors of functional genes. Both of these metrics were lower on average for the Class 1 scB copies than for Class 1 scA or for all other genes in the *Arabidopsis* genome. This was true whether looking at all Class 1 pairs combined or separated by duplication mechanism (Supplementary Figure 4), suggesting that Class 1 scB genes, as a group, are enriched for pseudogenes. Additionally, Class 1 pairs with the scB copy expressed in at least one SRA library have higher mean K_a_/K_s_ ratios than those of Classes 2–4 (Class 1: 0.65; Classes 2–4: ≤0.44), further suggesting enrichment for pseudogenes. Nonetheless, because mean Ka/Ks is below 1 for these Class 1 pairs, and lower than for the 7% of Class 1 pairs for which there is no evidence of scB expression in any SRA libraries (mean = 1.41, median = 0.92; *t* = −7.3, df = 148, *p* < 0.001), it is likely that at least some Class 1 scB genes expressed in SRA libraries are functional. Class 1 had the highest standard deviation in K_a_/K_s_ (Supplementary Table 6), consistent with this class comprising a mixture of pseudogenes and functional genes under different selective pressures.

In pairs comprising Classes 2–4, both paralogs are expressed in at least one cluster, with Classes 3 and 4 distinguished from Class 2 by biased paralog expression defined at the stringent threshold of 9:1, and Class 3 and 4 pairs differentiated by whether the bias was unidirectional, with only scA ever being dominant (Class 3), or whether the bias was reciprocal, with scA and scB dominant in different RCCs (Class 4; Table 1). Whereas the majority of Class 0 and Class 1 pairs are from proximal, tandem or young transposed duplicates, the majority of pairs in Classes 2–4 were produced by WGD or older transpositions (Figure 5). This pattern was most pronounced for Class 3 (Figure 5). Class 4 pairs comprised by far the smallest number of pairs (only 1.5–5.6% of pairs among duplication types), but showed the same pattern, with WGD and older transposed pairs having a larger percentage representation than the other SSD types.

**FIGURE 6:**
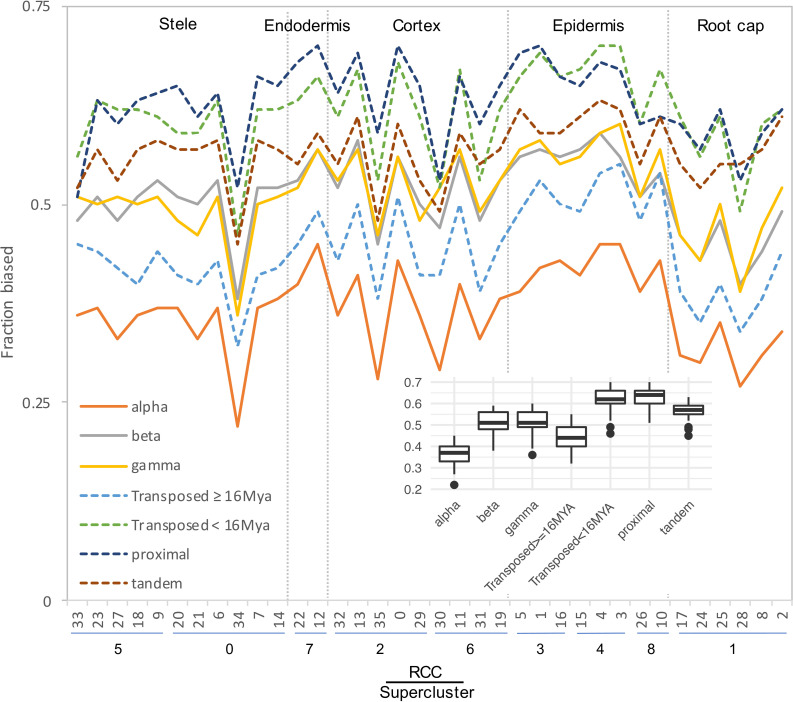
Fraction of gene pairs exhibiting paralog expression bias by RCC. RCCs are arranged by supercluster and ordered roughly from the interior (stele) to the exterior (epidermis) and tip (root cap) of the root. Each line indicates the fraction of gene pairs from the specified duplication mechanism that exhibit expression bias per RCC. For ease of comparison, whole genome duplications are shown with solid lines, and small scale duplications are shown with dashed lines. Individual gene pair by RCC combinations that lacked sufficient read depth to detect bias were excluded from the analysis. Inset, box plot summarizing the distribution of bias fractions by duplication type.

Expression classes aggregate data across all RCCs to summarize expression patterns, but do not provide information about paralog pair behavior in individual RCCs. Different duplication types also showed very different percentages of pairs exhibiting bias in individual RCCs, with proximal, tandem, and younger transposed classes all showing greater levels of bias than WGD and older transposed pairs (Figure 6). Homoeologs from alpha WGD pairs are the least likely to show expression bias. The fraction of biased pairs also varied by RCC, with root cap clusters generally exhibiting the least bias across all types of duplicates (Figure 6).

### Pairs Showing Extreme Reciprocal Paralog Expression Bias

Class 4 pairs are defined by extreme reciprocal expression biases (“reciprocal fixation”) of paralogs across clusters, and are the most likely cases of expression subfunctionalization following duplication. To determine the degree to which cell-level data provided greater resolution to detect reciprocal fixation, we tallied the number of Class 4 genes: (1) between bulk tissues (root vs. leaf SRA libraries); (2) across the 9 superclusters, roughly representing cell types; (3) across the 36 RCCs, further subdividing putative cell types into cell states; and (4) between individual cells of the 36 RCCs (Figure 1).

**FIGURE 7:**
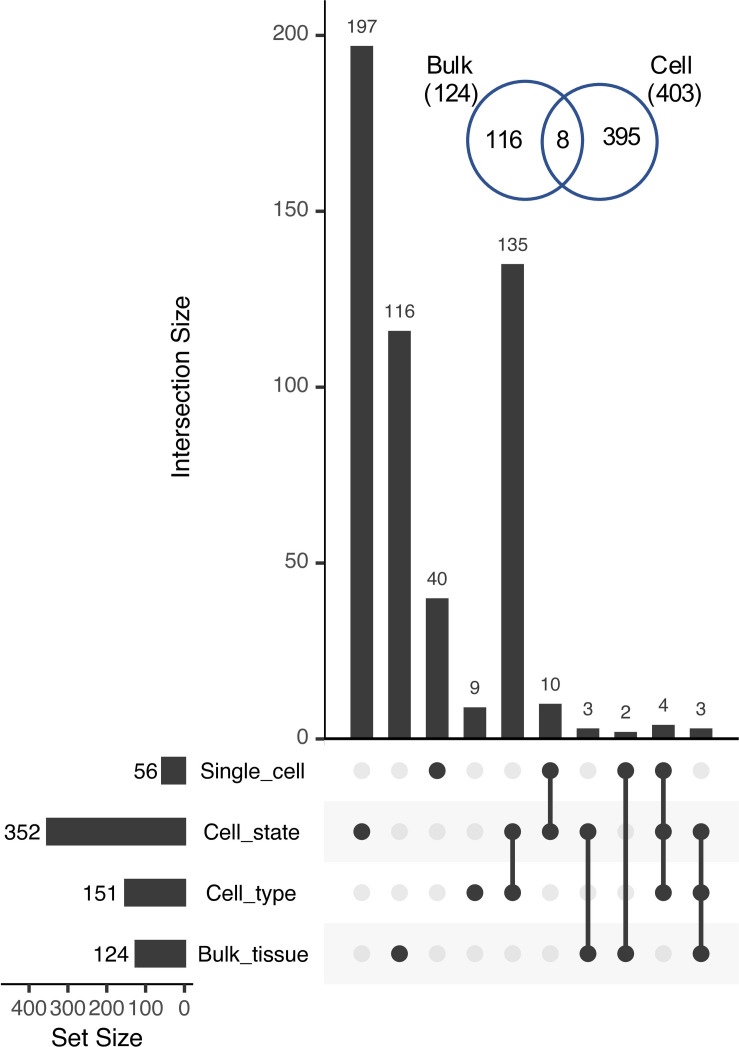
UpSet plot of intersections among Class 4 genes identified at different levels of organization. Inset, Venn diagram showing the overlap between Class 4 pairs identified in bulk tissue (Bulk) vs. those identified at sub-tissue levels (Cell; including cell type, cell state, and single cells).

The number of cases of reciprocal paralog expression bias identified using the single cell data was three times greater than those identified in the bulk tissue comparison (403 vs. 124; Figure 7). Most of these cases (352) were identified at the level of the 36 RCCs, suggesting that in root tissue, expression subfunctionalization occurs more frequently among cell states within a cell type than among cell types, with the caveat that the 9 superclusters may include more than one cell type, and that such heterogeneity could reduce the estimated number of Class 4 pairs.

Within the 9 superclusters, only 56 gene pairs exhibited reciprocal fixation at the level of single cells (an over-representation of cells exhibiting significant bias favoring one copy in some cells, and the other copy in other cells, with few or no cells co-expressing both equally). This is likely an undercount of the true number, however, due to the low read count per cell. Of the 87.5 million possible cell × gene pair comparisons (7,519 cells × 11,631 gene pairs), 19.2 million had non-zero read counts, but of these, 17.5 million had counts that were nonetheless too low to detect bias at our threshold of 9:1.

In contrast to 56 significantly non-overlapping gene pairs, 1,363 gene pairs exhibited significant overlap at the level of single cells (cells expressing one copy were significantly more likely to express the other copy as well). Thus, at the single cell level, paralogs appear to be coexpressed 24.3-fold (1,363/56) more often than not. Alpha WGD duplicates were the most likely to exhibit significant overlap at the level of single cells, whereas gamma WGD duplicates were the most likely to exhibit significant non-overlap (Table 2).

**TABLE 2:** Counts of overlap and non-overlap of paralog expression in individual cells.

Duplication type	Overlap	% of total	Non-overlap	% of total
alpha	593	18.64%	13	0.41%
beta	170	11.72%	5	0.34%
gamma	64	12.28%	4	0.77%
Transposed ≥ 16 MYA	245	13.16%	13	0.70%
Transposed < 16 MYA	103	6.06%	7	0.41%
proximal	39	4.97%	2	0.26%
tandem	149	7.00%	12	0.56%

Notably, Class 4 pair genes at all four levels of organization (bulk tissue, cell types, cell states, and single cells) are enriched for extracellular functions (e.g., extracellular region, apoplast, cell-cell junction), and each is also enriched for some aspect of the cell periphery (e.g., cell wall, plasma membrane) (*p* < 0.05; Holm-Bonferroni; Supplementary Table 3). Thus, reciprocal fixation of expression, suggestive of partitioning of function, appears to occur preferentially among paralogs functioning at the cell surface. Beyond this commonality, however, GO enrichment analysis suggests that paralogs exhibiting extreme reciprocal fixation at the bulk tissue level differ functionally from those exhibiting reciprocal fixation at finer levels of resolution. At the bulk tissue level, Class 4 pair genes are preferentially involved in lipid metabolism and vesicle trafficking (exocyst), whereas at the supercluster, RCC, and single cell levels, Class 4 pair genes are preferentially involved in cell wall modification (e.g., cell wall organization or biogenesis, hemicellulose metabolic process) and response to stress (e.g., response to oxidative stress, response to toxic substance; Supplementary Table 3).

### Concerted Divergence of Paralogous Genes

Overall, WGD gene pairs were more likely to exhibit reciprocal fixation across the 36 RCCs than were SSDs (*χ*^2^ = 22.1, *p* < 0.001). In total, 195 out of 5,018 WGD pairs (3.9%) were assigned to Class 4, compared to 157 out of 6,449 SSD pairs (2.4%).

It has been proposed that WGD facilitates functional differentiation by simultaneously duplicating entire gene networks, thereby providing the raw material for “concerted divergence” (Blanc and Wolfe, 2004) of whole biological pathways. We looked for evidence of this phenomenon, focusing on the genes duplicated by the alpha WGD. Out of a total of 3,096 alpha gene pairs, 176 genes in 88 pairs belonged to Class 4, the class most consistent with expression subfunctionalization within roots.

**FIGURE 8:**
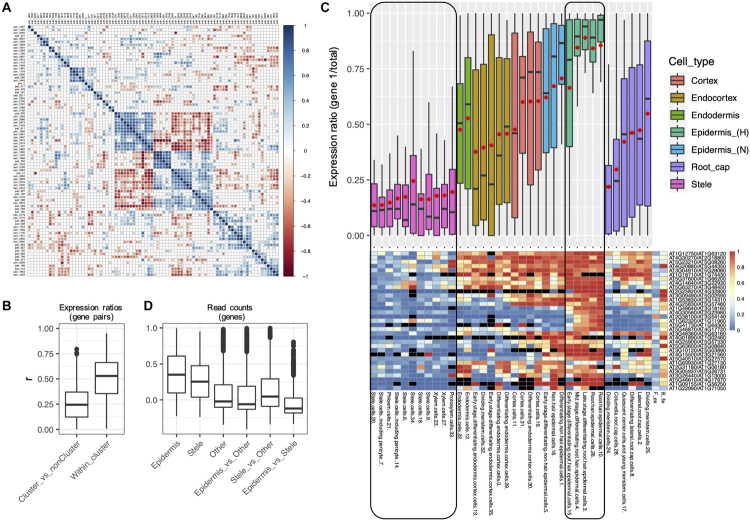
Evidence for concerted divergence among alpha homoeologs. **(A)** Correlogram of pairwise correlation coefficients of expression ratios for all 84 alpha Class 4 gene pairs. Each row or column represents a single gene pair. Significant positive pairwise correlations (scA from both pairs show similar patterns of dominance/non-dominance) are shown in blue, and significant negative correlations (scA from one pair shows a similar pattern as scB from the other pair) are shown in red. Non-significant correlation coefficients are shown in gray. A cluster of 33 significantly correlated pairs is visible in the middle of the plot, in which each pair has diverged in expression in a correlated manner with each other pair, suggesting concerted divergence. **(B)** Pairwise correlation coefficients (r) of homoeolog expression ratios within the cluster of putative concerted divergence (Within cluster), and between this cluster and all other Class 4 alpha pairs (Cluster_vs_nonCluster). **(C)** Boxplot and heatmap showing the distribution of expression ratios (scA/total) for the 33 gene pairs comprising the largest cluster of alpha gene pairs exhibiting putative concerted divergence. Expression ratios are shown for each of the 36 RCCs (cell states), and these are clustered and color coded by cell types. A common color was assigned to the two “Stele” superclusters and to the two “Epidermis (H)” superclusters to emphasize the partitioning of expression between these cell types. Red points in the boxplot indicate mean values. Heatmap colors indicate raw expression ratios (gene 1/total) without application of Wilson tests. **(D)** Pairwise correlation coefficients of read counts by RCC among the homoeologs within the putative cluster of concerted divergence showing epidermal bias (Epidermis) or stele bias (Stele), for all other Class 4 alpha genes (Other), and between each of these gene sets.

For each of these 88 Class 4 alpha WGD pairs we calculated an expression ratio (scA/total) for each of the 36 RCCs. Then, for each alpha pair, we calculated correlation coefficients with every other alpha pair based on these 36 expression ratios. To the extent that two alpha pairs have diverged in concert, we would expect their expression ratios to be either positively correlated (*r* > > 0) if scA from both pairs are coevolving, or negatively correlated (*r* < < 0) if scA from one pair is coevolving with scB from the other pair. For alpha pairs that are diverging independently of each other, we expect to see no correlation (*r* = 0). By this approach, 33 of the 88 pairs formed a distinct set with significantly correlated expression ratios (*p* < 0.05; Figures 8A,B), suggesting concerted divergence into two separate networks. Several additional, smaller clusters were evident as well.

Within the 33 pair cluster, paralogs have diverged in expression such that one copy of each pair is preferentially expressed in the stele and the other copy is preferentially expressed in the epidermis (Figure 8C). Paralogs exhibited a range of biases in intervening layers. The stele-dominant copy of each pair is also dominant in columella cells and one population of meristematic cells in the root cap, whereas the epidermis-dominant copy is weakly dominant in a second population of dividing meristematic cells. As expected, pairwise correlation coefficients of expression profiles were higher within the two diverged gene sets (epidermis-biased and stele-biased) than among the Class 4 alpha pairs not in either gene set, or between the epidermal and stele gene sets (Figure 8D), consistent with the possibility that the two gene sets have diverged in concert and are acting as separate functional modules.

The 66 genes in the cluster are not enriched for any GO terms or protein domains, though several genes are involved in calcium signaling. Similarly, neither set of 33 genes in the two separate co-expressed networks is enriched for GO terms, and both homoeologs have equivalent annotations in most cases. Thus, there is no obvious functional differentiation discernable at the level of gene ontology between homoeologs in the two networks.

However, 13 genes in the epidermal network have protein-protein interactions annotated in the InTact database^2^, and two of these genes encode proteins that interact directly: a MYB transcription factor encoded by AT2G31180 and a calcium-sensing calmodulin protein encoded by AT4G14640. A third gene (AT4G30560) encodes a calmodulin-regulated ion channel that interacts indirectly with the calmodulin protein via a protein kinase intermediary (AT4G04570), suggesting a gene module involved in calcium-dependent ion trafficking functioning in the epidermis. No equivalent interactions are annotated among the stele-biased genes, and no genes from the epidermally-biased co-expression network directly interact with genes from the stele-biased network. Collectively, these observations suggest that the two homoeologous networks have diverged in concert, both spatially and functionally.

### Correspondence of RCC Expression Classes With Functional Divergence of Paralogs

Expression and function are generally synonymized in discussions of gene evolution (e.g., Panchy et al., 2019). For gene pairs with biased paralog expression profiles, we therefore assessed the correspondence of different expression patterns with other measures of functional differentiation.

**FIGURE 9:**
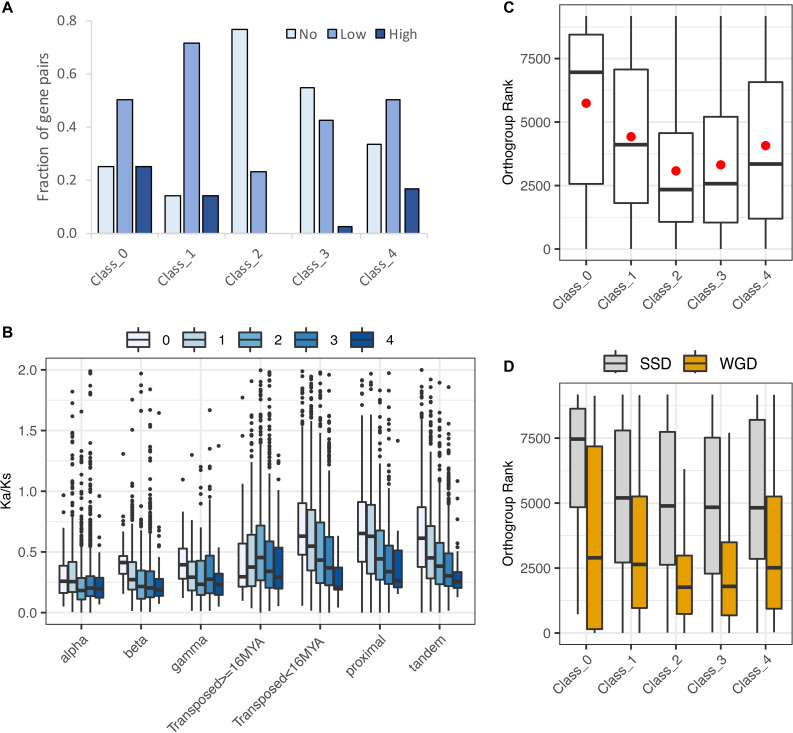
Evidence for functional divergence and gene dosage sensitivity. **(A)** Levels of morphological divergence (Hanada et al., 2009) by expression class. **(B)** K_a_/K_s_ by duplication mechanism and expression class. K_a_/K_s_ values were cut off at 2 for clarity of presentation, but extended beyond this value in all cases. **(C)** Correspondence of expression classes with dosage sensitive gene families (“orthogroups”: Tasdighian et al., 2017). Lower orthogroup rank indicates greater overall dosage sensitivity. Orthogroup rank by expression class for 5,387 duplicate pairs. **(D)** Orthogroup rank by expression class separately for WGD and SSD duplication types.

Hanada et al. (2009) categorized 492 *Arabidopsis* gene pairs as showing low, medium, or high morphological diversification on the basis of knockout phenotypes for one or both paralogs. Of these, only 94 were included among the Wang et al. (2013) duplicate pairs, of which one or both copies were expressed in roots for 90 (Supplementary Table 3). Despite the small number of pairs in both data sets, the distribution of pairs exhibiting or not exhibiting some degree of morphological variation differed significantly by expression class (*χ*^2^ = 12.1, *p* = 0.02). Unlike all other classes, the majority of Class 2 pairs (no biased paralog expression) exhibited no morphological divergence, and no Class 2 pairs exhibited high morphological divergence (Figure 9A). If this small sample is representative, the results suggest that pairs of genes with unbiased paralog expression can tolerate the loss of expression from one paralog, suggesting that the two paralogs are not currently maintained either by dosage constraints or by essential functional differences between the two paralogous proteins.

In contrast to Class 2, only around 10% of Class 1 pairs were in the no morphological diversification category, suggesting that paralogs of Class 1 pairs in which the non-root paralog is not a pseudogene have diverged in function. Classes 3 and 4 were intermediate between Classes 1 and 2 (Figure 9A). Like Class 1, Classes 3 and 4 both partition paralog expression, but in these classes partitioning is within the root, between different RCCs. Shared root expression could suggest less functional differentiation between paralogs; moreover, many pairs have both paralogs expressed in RCCs other than those fixed for one paralog, making the pair’s behavior in those RCCs more like Class 2. The higher proportion of Class 4 pairs showing some degree of morphological diversification suggests that reciprocal fixation – the near-exclusive use of a different paralog in different RCCs – may be more indicative of a shift in function between paralogs than consistent dominance of one paralog (Class 3).

The ratio of replacement to silent nucleotide substitutions (K_a_/K_s_) is understood to be a measure of functional divergence. For all duplication types and expression classes, K_a_/K_s_ distributions were strongly skewed, with median and mode less than 1.0 (Figure 9B and Supplementary Tables 4, 6), indicating that most gene pairs are evolving under purifying selection, as expected. As reported by Qiao et al. (2019) for 141 phylogenetically diverse plant genomes, average K_a_/K_s_ varied with duplication type, with WGD classes showing lower K_a_/K_s_ than tandem, proximal, and young transposed pairs. Variances among both duplication types and expression classes differed significantly (*p* < 0.001), suggesting differences in the number of pairs showing positive selection or containing an expressed pseudogene. Class 1 had the highest standard deviation in total and for all duplication types except proximal SSDs, suggesting that this class includes either pseudogenes, genes under positive selection, or both. Class 4 had the smallest standard deviation (Supplementary Table 6). The large variance for Class 1 is also consistent with its having the highest percentage of morphologically divergent paralog pairs as classified by Hanada et al. (2009).

The fact that Class 4 has the lowest average K_a_/K_s_, and the smallest variance around the mean seems to indicate uniformly strong purifying selection. The relatively low level of amino acid replacements between Class 4 paralogs, coupled with the functional divergence (see above), suggests that these pairs may be diverging in their promoter regions (sequence and/or accessibility).

### Dosage Sensitive Gene Families

Many duplicate gene pairs are thought to be preserved not by functional divergence, but to maintain stoichiometric relationships in protein complexes and interacting networks following whole genome duplication; the same constraints lead to the loss of paralogs following SSD (e.g., Freeling, 2009; Birchler and Veitia, 2010, 2012, 2014). Tasdighian et al. (2017) ranked 9,178 core gene families (“orthogroups”) across 37 angiosperm based on how strongly paralogs have been retained in duplicate following WGD vs. SSD; gene families with high levels of WGD retention and minimal SSD retention are defined as “reciprocally retained,” and strong reciprocal retention is considered a hallmark of selection to maintain dosage balance. 6,308 Wang et al. (2013) pairs occur among these 9,178 orthogroups; 853 of these pairs had paralogs assigned to two different orthogroups, and an additional 68 were not assigned to expression class because one gene model was obsolete; these were excluded, leaving 5,387 pairs with one or both members assigned to an orthogroup and class (Supplementary Table 3). We divided the 5,387 pairs by expression class and determined the “orthogroup rank” as in Tasdighian et al. (2017), wherein orthogroups with the highest reciprocal retention are ranked the highest (i.e., smallest numerical rank). Class 2 pairs were the most dosage-sensitive, significantly more highly ranked than other classes (Class 2 vs. Class 3: *z* = −2.22, *p* = 0.029; Dunn’s test of multiple comparisons with Benjamini-Hochberg correction; all other comparisons significant at *p* ≤ 0.0001; Figure 9C). This is consistent with the expectation that if dosage balance is responsible for preserving both paralogs, neither paralog is likely to be strongly biased in its expression. Other classes showed less evidence of dosage-based constraints, with Class 1 pairs showing the least effect (highest orthogroup ranks), consistent with its paralogs being expressed in different organs. Although Class 3 and 4 pairs have some RCCs fixed for one paralog, other clusters have both paralogs expressed, so it is possible that dosage balance is necessary in some cell types but not in others, where divergence of expression pattern (“function”) is occurring.

Dividing the 5,387 pairs into WGD and SSD duplication types (Figure 9D) showed the expected result that SSD pairs are less affected by dosage constraints than are WGD pairs. Alpha WGD duplicates showed the same overall pattern of classes as did the full dataset and the WGD duplicates combined (data not shown).

### Expression Patterns of Duplicate Pairs by Subgenome

The loss of duplicated genes following polyploidy – fractionation (Langham et al., 2004) – is often unequal across homoeologous subgenomes, and such biased fractionation is thought to begin with unequal expression of homoeologs (e.g., Steige and Slotte, 2016). Schnable et al. (2012) assigned *Arabidopsis* homoeologs from 817 *Arabidopsis* alpha WGD pairs to two putative homoeologous subgenomes, A and B, with A being the dominant subgenome, characterized by lower rates of gene loss and higher expression of remaining genes. We compared subgenome assignments with our scA/scB classification based on expression of Wang et al. (2013) alpha WGD homoeologs in RCCs, and found representation of pairs from all our classes, with 9 Class 0 pairs, 82 Class 1 pairs, 240 Class 2 pairs, 377 Class 3 pairs, and 23 Class 4 pairs (86 pairs from Schnable et al. (2012) were not included in the Wang et al. (2013) assignments; Supplementary Table 3). We wished to determine whether the paralog with higher expression in RCCs most commonly was from the dominant subgenome. For this, Class 0 (no root expression) and Class 2 (no extreme expression bias) pairs were irrelevant, whereas Class 1 may have bias, and Classes 3 and 4 must have at least one biased context. Of the 453 pairs in the Schnable et al. (2012) assignments for which we did see bias in one or more contexts (Supplementary Table 3), the scA/scB assignments based on dominance observed in our expression data were consistent with the A/B subgenome dominance assignments 261 times, which is significantly more than expected by chance (*p* = 0.0007; binomial test). Of the three classes, Class 1 was most concordant, with approximately 4 times the number of pairs agreeing than disagreeing, while Classes 3 and 4 both had roughly only 1.25 times more cases of agreement than disagreement, again a statistically significant difference (*p* = 0.003; Fisher’s exact test). Though the majority of Class 1 scB paralogs are expressed in other tissues (Supplementary Table 5), they are expressed at lower levels and under fewer conditions than their scA counterparts (Supplementary Figure 4). Their over-representation in the non-dominant subgenome, therefore, is consistent with the hypothesis that genome dominance (fractionation bias) is driven by expression level bias. The average F_ex_ value for pairs in agreement with the subgenome assignments was 0.36 compared to 0.31 for those in disagreement.

We also asked if the 33 alpha pairs representing potential concerted divergence (Figure 8) have partitioned expression by subgenome. The stele-dominant set includes eight homoeologs from subgenome A and five from subgenome B, and the epidermis-dominant set includes five homoeologs from subgenome A and seven from subgenome B. 41 of the 66 genes in the two sets genes were not assigned to subgenome by Schnable et al. (2012). This lack of subgenome assignment for most genes makes it difficult to assess patterns of subgenome partitioning, but the mixed representation in each pathway suggests that the two sets most likely diverged in concert after the alpha WGD, enlisting genes from both subgenomes, rather than in the progenitors of the polyploid.

### Shared and Unique Transcriptomes of Cell Clusters and the Evolutionary Polarity of Duplicate Pair Expression

The 22,669 genes that are expressed in at least one of the 36 RCCs include 7,653 genes, comprising 33.8% of the overall root transcriptome and well over half of the transcriptomes of some RCCs, that are expressed in all 36 root cell clusters (RCC-u genes; Figure 4 and Supplementary Table 3). We found that over 99% of RCC-u genes are expressed in at least one non-root SRA dataset (data not shown). Among genes expected to belong to the RCC-u class are genes that are expressed in all cells of the plant (both root and non-root), as well as genes expressed in all root cells but not in all cell types of other plant tissues. A total of 3,505 RCC-u genes (45.7% of RCC-u genes) were among the set of 4,577 genes expressed in all 11 *Arabidopsis* tissues studied by Cheng et al. (2017) Thus, nearly half of RCC-u genes may be ubiquitously expressed in plant cells.

RCC-ubiquitous genes are thus an interesting class to consider because their presence in all RCC transcriptomes suggests that their expression is indispensable in all root cell types and states, and, for over 45%, perhaps in all cells. This suggests that prior to any duplication – whether WGD or SSD – each of these genes was expressed as a single copy in all RCCs, and therefore that immediately after duplication both paralogs were RCC-u. Under these assumptions, pairs in which only one paralog is ubiquitously expressed in root cells are inferred to represent evolutionary losses of expression of the non-RCC-u paralog, rather than gain of expression in some RCCs leading to ubiquitous expression of the RCC-u paralog. We further assumed that, immediately following duplication, the two paralogs were expressed at similar levels.

Among the 7,653 RCC-u genes are 4,538 genes belonging to 4,639 Wang et al. (2013) gene pairs (a gene can belong to more than one pair due to nested duplications). 40 of these gene pairs include one gene that is RCC-u and one that is an obsolete gene model; consequently, these pairs are not assigned to an expression class, leaving 4,599 Wang et al. (2013) RCC-u gene pairs assigned to an expression class. Only 1,552 pairs retained both genes as RCC-u, and among these, only 847 pairs did so without significant expression bias (Class 2; Table 3). Thus, both copies retain the putative ancestral expression profile for only 18.4% (847/4,599) of RCC-u pairs. 705 pairs retained both genes as RCC-u but showed biased expression in one or more RCCs (Classes 3 and 4). The remaining 3,047 pairs include one copy that is RCC-u and one that is not, indicating changes in expression in at least one copy from the ancestral profile. For 327 pairs, these shifts were subtle, as we did not detect significant bias (Class 2). In the remaining cases (2,720 pairs), the shift from the hypothesized ancestral condition of both paralogs being RCC-u was more dramatic, including the 505 pairs for which the second paralog was not expressed in any root cell type (Class 1; Table 3).

**TABLE 3:** Breakdown by expression class of 4,599 Wang et al. (2013) paralog pairs ubiquitously expressed in root cell clusters (RCC-u gene pairs).

Class	Count	RCC-u mixed	RCC-u both
0	0	0	0
1	505	505	0
2	1,174	327	847
3	2,780	2,126	654
4	140	89	51

These counts are further broken down by duplication mechanism in Supplementary Table 8. For alpha duplicates, there were 1,685 pairs with one or both genes RCC-u. Of these, in 294 pairs both homoeologs were RCC-u but show significant bias in at least one cluster (17.4%; Classes 3 and 4), and 949 pairs (56.3%) have only one RCC-u copy with varying degrees of bias in clusters where both homoeologs are expressed (Supplementary Table 8). Conversely, 442 pairs (26.2%) show no evidence for shifts from the ancestral state (both copies are RCC-u with no bias [Class 2]). This is the highest fraction of pairs with both copies retaining the ancestral expression pattern of any duplication type. Gamma and beta WGD duplicates exhibited the next highest fractions (16 and 15.1%, respectively), and the SSD duplicates had the lowest. This suggests that WGD duplicates are more constrained to retain their ancestral expression patterns, perhaps due to dosage constraints.

Representation in the RCC-u class itself also varied with duplication type, with over-representation of WGD duplicates expressed across all RCCs relative to the total fraction of WGD duplicates in the genome (Supplementary Figure 5). In contrast, SSD duplicates, other than older transposed duplicates, were under-represented relative to their representation in the genome as a whole, more like genes lacking a duplicate in the Wang et al. (2013) set and for which one paralog has presumably been lost after duplication (“singletons” in Supplementary Figure 4).

Evolutionary shifts in expression of paralog pairs were also observed among the remaining 59.6% of pairs comprising the cumulative root transcriptome that are expressed in 1–35 RCCs (non-RCC-u pairs). Their lack of ubiquitous expression in root cell types/states makes it more difficult to determine whether expression of a given gene in a particular RCC represents the ancestral state or the derived state, and thus to hypothesize the ancestral condition for a paralog pair with only one member expressed in an RCC. If we assume that at the time of a duplication, both paralogs retained the expression pattern of the single copy gene progenitor (see below for discussion of this assumption), then pairs with unbiased expression of both paralogs or neither paralog in a given RCC are most readily explained as retaining the expression state of their single copy progenitor, since two independent gains or losses of expression would need to be hypothesized otherwise.

Pairs with only one paralog expressed in any RCC (Class 1), or with biased expression of one paralog (Classes 3 and 4), could equally parsimoniously be inferred to have gained or lost expression of one paralog in RCCs for which the pair shows fixation. In either case, however, an evolutionary shift in expression from the inferred ancestral condition is involved. The total number of RCCs showing fixation of a paralog pair in the non-RCC-u class (i.e., the number of red or blue cells in a heatmap of non-RCC-u pairs, similar to the examples shown in Figure 2) was 22,897; this was 45% of the total number of number of heatmap cells (non-RCC-u paralog pairs × 36 RCCs; Supplementary Table 9).

### Expression Differences Among and Within Cell Types and Cell States

In addition to assessing the behavior of gene pairs following their duplication by WGD or SSD, we were interested in exploring differential responses of different cell types/states to gene duplication. The 9 superclusters identified by Ryu et al. (2019) in some cases aggregated known cell types (e.g., Ryu supercluster 1, in which cells of the quiescent center are grouped with root cap cells; also Ryu superclusters 0, 2, and 5). These presumably artificial superclusters were disaggregated in the 36 RCC data studied here (e.g., Figures 2, 3, 6). This resulted in the identification of some extreme reciprocally biased expression patterns between RCCs of the Ryu et al. (2019) superclusters (Figure 6). For example, there were 32 reciprocal fixations within Ryu supercluster 5, with protoxylem cells being particularly differentiated in their expression pattern relative to other cell types grouped in the same supercluster.

Ryu et al. (2019) studied the developmental trajectories of root hair cells, as well as other cell types, and those cell developmental states are also included in the 36 RCC data, allowing us to search for differential responses to duplication among developmental states of more confidently identified cell types: non-hair epidermal cells (three RCCs of supercluster 3), root hair epidermal hairs (three RCCs of supercluster 4), late stage and mature cortex cells (four RCCs of supercluster 6), mature endodermis cells (two RCCs of supercluster 7), and a second root hair supercluster (two RCCs of supercluster 8). No cases of reciprocal fixation were found among states belonging to superclusters 4, 7, or 8, but a beta WGD pair (AT3G13750/AT5G56870) was found to be reciprocally fixed for states within both superclusters 3 and 6; the alpha pair AT3G18950/AT1G49450 (row 12 in Figure 2A) and the young transposed pair AT5G39580/AT5G64100 were found to be reciprocally fixed for states within supercluster 6.

In addition to the small number of reciprocal shifts in extreme paralog expression bias among states of the same cell type, there were many other instances where the expression patterns of paralog pairs were not homogeneous across states of a given homogeneous cell type (e.g., rows 1, 2, 7, 8, 11, and 12 in Figure 2A). For example, there were 87 alpha WGD pairs that had no expression of either paralog in one RCC of non-hair epidermal cell supercluster 3, fixation of one paralog in a second RCC of the supercluster, and balanced expression of both paralogs in the third RCC (Supplementary Table 7). Overall, for supercluster 3, 35% of alpha WGD pairs (1,119/3,181) had non-homogeneous expression across its three RCCs; for all duplication types, the average was 30% for this supercluster. In all five of these putatively homogeneous cell type superclusters, the three WGD classes and the older transposed duplication class had higher percentages of non-homogeneous expression; the actual percentages were roughly correlated with the number of RCCs in a supercluster – finer division resulted in greater heterogeneity – but superclusters with the same number of RCCs differed from one another (e.g., superclusters 3 and 4, each with three RCCs, had overall heterogeneity percentages of 30 and 23.3%, respectively), suggesting cell type-specific patterns of paralog expression during differentiation.

**FIGURE 10:**
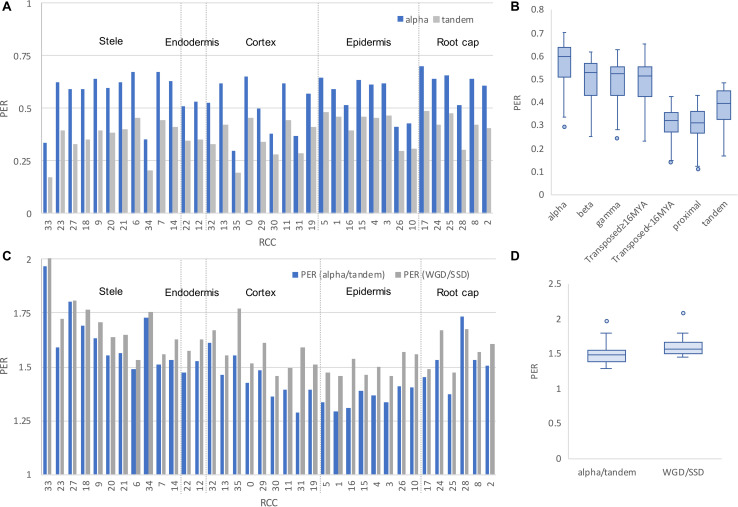
Paralog retention ratio (PER) by RCC and duplication mechanism. **(A)** PER for alpha and tandem duplicates by RCC. The grouping of RCCs by cell type is indicated with dashed lines. **(B)** PER distributions across RCCs by duplication type. **(C)** Differential retention ratios for WGD/SSD and alpha/tandem duplication types. **(D)** Distribution of differential retention ratios (alpha/tandem and WGD/SSD).

To explore differential responses of RCCs to different types of gene duplication, we calculated a “paralog expression retention” (PER) index, defined as the number of pairs expressing both paralogs in a given RCC divided by the number of pairs with at least one paralog expressed in the RCC, and compared this value across RCCs for all pairs except those for which both paralogs were classified as RCC-u. There was significant variation in PER by both RCC (*χ*^2^ = 4285.8, df = 35, *p* < 0.001) and duplication type (*χ*^2^ = 615.1, df = 6, *p* < 0.001), with WGD and older transposed duplicate pairs showing higher rates of paralog retention (Figures 10A,B). Variation in PER across RCCs was likely due, in part, to differences in sequencing depth per RCC (RCCs with fewer total reads are more susceptible to dropout). On the assumption that sequencing depth has the same relative effect on PER for different duplication mechanisms in all RCCs, we adjusted for differences in sequencing depth by calculating the differential retention ratio, PER_wgd_/PER_ssd_ for each RCC. Because of our specific interest in the alpha WGD we also calculated PER_alpha_/PER_tandem_ (Figure 10C). These two ratios were similar (Figure 10D). Variation in differential retention ratio across RCCs is likely due to differences in cell biology that reflect the degree to which the genes whose expression comprises the transcriptome are dosage sensitive; higher PER of WGD pairs is expected given their greater representation among dosage sensitive gene families (Tasdighian et al., 2017; Qiao et al., 2019).

## Discussion

Against the backdrop of constant and ongoing single gene duplications (Lynch and Conery, 2000), the genome of *A. thaliana* bears the legacy of multiple polyploidy events, dominated by the alpha WGD event, which phylogenomic studies indicate took place 32–43 MYA (Edger et al., 2018). The availability of single cell transcriptome data from *A. thaliana* roots (Ryu et al., 2019) allowed us to assay the effects of this event on gene expression in unprecedented detail, from the dual perspectives of the genes duplicated by the polyploidy event and of cell types that, after millions of years of existence at a lower ploidy, were suddenly presented with the challenges and opportunities of a doubled genome.

### Expression Evolution of Alpha WGD Homoeolog Pairs in Root Cell Types and Cell States

For simplicity we have assumed, as others have done (e.g., Blanc and Wolfe, 2004; Panchy et al., 2019) that most alpha WGD pairs initially conserved their ancestral functions and partitioned their expression equally between what are now their two homoeologs. This is a questionable assumption if, as is widely accepted based on biased fractionation of the homoeologous genomes of *Arabidopsis* (Schnable et al., 2012; Woodhouse et al., 2014; Cheng et al., 2018), the duplication was an allopolyploidy event, merging two already differentiated genomes. Although root cell types presumably were conserved between these progenitor species, their transcriptomes no doubt differed from one another, given the effects of mutation and drift even under stable selection (Lynch, 2020). For example, fiber cell transcriptomes differ between the diploid species whose genomes merged to form tetraploid cotton (Yoo et al., 2013; Gallagher et al., 2020). Thus, differences in the expression patterns of alpha WGD homoeologs may be due to “parental legacy” (Buggs et al., 2014; Steige and Slotte, 2016) and not solely to genome duplication. We see some evidence of this in the correlation of our biased expression classes with the homoeologous subgenomes. This is particularly true of Class 1 pairs, many of which may include pseudogenes as the more weakly expressed homoeolog (scB). In the 82 alpha WGD Class 1 pairs mapped to subgenomes, we found that scB was four times more likely to be on the more highly fractionated subgenome B.

Nevertheless, it seems likely that, on average, more of the differentiation of alpha WGD homoeologs has occurred subsequent to genome merger, for three reasons. First, the time since polyploidy is at least 3× longer than the time since speciation prior to allopolyploidy (Edger et al., 2018), so even if homoeolog divergence has been proportional to time, more differentiation would be expected to have taken place in the last 30 MY than in the first 10 MY since progenitor speciation. But, second, genome evolution after allopolyploid merger and duplication is thought to be far from the clock-like divergence that might be expected after diploid speciation, beginning with an initial “genomic shock” phase of rapid genetic and genomic change (McClintock, 1984), followed by diploidization that returns the allopolyploid to a more conventional evolutionary rate (Wendel, 2015). Finally, orthologs generally evolve conservatively with respect to function and expression pattern, at least relative to paralogs (Gabaldon and Koonin, 2013).

Even with the simplifying assumption of initially shared expression patterns, however, determining whether the expression of only one paralog in an RCC is the ancestral or the derived state required a second assumption, as no close outgroup is available (e.g., the closest relative to an alpha duplicate pair generally is a beta WGD homoeolog, diverged around 100 MY from either alpha homoeolog; Panchy et al., 2019). We thus focused on gene pairs where at least one member of an alpha homoeolog pair was ubiquitously expressed in all root cell clusters (i.e., pairs that contained at least one RCC-u gene, detectable in all 36 RCCs), reasoning that these genes were likely to be necessary for the functioning of all cell types and cell states in roots. If so, the ancestral condition of the pre-alpha WGD single copy gene was ubiquitous expression in root cells, and the ancestral condition of the two alpha WGD paralogs was equal expression in all RCCs.

With these two assumptions, we looked for departures from the ancestral condition, representing shifts in expression following homoeolog divergence from their common ancestor. Out of 1,685 alpha pairs with at least one RCC-u homoeolog, we found 949 with only one RCC-u homoeolog, including 94 pairs with only one homoeolog expressed in roots (Class 1 alpha WGD pairs; Supplementary Table 8). Additionally, we observed 294 alpha pairs with both homoeologs RCC-u, but with biased expression of one or both homoeologs in one or more RCCs (280 Class 3 and 14 Class 4 pairs, respectively). Thus, in 73.8% of RCC-u alpha WGD pairs, at least one member has changed its expression as homoeologs have diverged.

This logic does not apply to non-RCC-u pairs – those with neither member expressed ubiquitously across the 36 RCCs. However, even without being able to hypothesize the polarity of the change, under the assumption that both paralogs initially were expressed in an unbiased fashion in the same cell immediately after their duplication, we can assess the amount of change, and we found that 72% of alpha WGD non-RCC-u pairs had one homoeolog uniquely or preferentially expressed in at least one RCC. High levels of expression differentiation between homoeologs are not unexpected. For example, in the older (ca. 60 MY) polyploid, *Gossypium raimondii*, over 90% of homoeologs show evidence of sub- or neofunctionalization (Renny-Byfield et al., 2014).

Determination of expression patterns at the level of root cell types/states also provided information on mechanisms of gene retention. The 590 alpha WGD RCC-u gene pairs that express both copies without strong bias (Class 2 RCC-u pairs; Supplementary Table 8) are candidates for preservation by dosage balance constraints (Papp et al., 2003; Coate et al., 2016; Tasdighian et al., 2017; Song et al., 2020). More broadly, all 957 alpha WGD Class 2 pairs, including those that are not expressed in all 36 RCCs (non-RCC-u) are also candidates for preservation by dosage balance. Consistent with this, Class 2 alpha WGD genes showed less evidence of functional divergence (Figure 9A) than did other classes, were more likely to belong to dosage-sensitive gene families as defined by Tasdighian et al. (2017; Figures 9C,D), and, as expected, showed less evidence of positive selection than did SSD pairs or alpha pairs in other expression classes (Figure 9B).

Alpha WGD pairs belonging to Classes 3 and 4 provide examples where homoeologs could be maintained by sub- or neofunctionalization. The polarity of expression shifts of the 1,570 alpha WGD Class 3 pairs is unknown, but each includes at least one RCC where there is novel bias subsequent to the divergence of the two homoeologs. This could occur by enhanced expression of one homoeolog or its recruitment to that RCC, the latter being consistent with neofunctionalization. Alternatively, bias could be achieved by diminished expression of the now more weakly-expressed homoeolog (consistent with subfunctionalization). For Class 3 pairs we do not know whether scB is the predominantly expressed paralog in any non-root cell type, which would likely be true if scB is also protected from loss by being essential. For Class 4 pairs, however, there is reciprocal fixation of the two paralogs of 88 alpha WGD pairs (nearly 3% of all alpha pairs) among the 36 RCCs, providing self-contained examples consistent with subfunctionalization (Supplementary Table 8) just within root cell types, novel information not available from bulk tissue samples (Figure 7). That divergence in expression pattern among these 88 pairs may be functional is suggested by our finding evidence for concerted divergence (Blanc and Wolfe, 2004) of networks between stele and epidermal cells (Figure 8).

### Responses of Cell Types to the Alpha WGD Event

The basic cell types of the root – xylem, phloem, epidermis, cortex, root hairs, root cap, etc. – are conserved across most plants that have roots, and presumably evolved early in the history of vascular plants (Raven and Edwards, 2001; Kenrick and Strullu-Derrien, 2014; Huang and Schiefelbein, 2015). Immediately following each of the three polyploidy events detectable in genomes of most Brassicaceae, each cell type must also have functioned well enough, through all of the developmental states leading to its mature condition, to allow the plant to survive.

Individual *Arabidopsis* root cell types and states comprising the 36 RCCs evolved their current expression patterns over 30–40 MY of speciation, divergence, and diploidization since the alpha WGD event, each expressing a subset of the homoeologs retained in the genome. Many of the nearly three quarters of the 1,685 alpha WGD RCC-u gene pairs showing evidence of evolutionary shifts in expression in at least one cell type or state (Supplementary Table 8) showed such shifts in many or all RCCs (e.g., given adequate statistical power, a Class 1 RCC-u pair would show an expression shift in each of the 36 clusters). Overall, we found that for both RCC-u and non-RCC-u alpha WGD pairs, over one third of all cell type/state × homoeolog pair combinations for which statistically robust measurements could be made showed evidence of expression shifts (Supplementary Table 9).

Moreover, each of the 36 cell types and states has had its own characteristic response to gene and genome duplication. For example, excluding RCC-u genes, approximately 60% of alpha WGD pairs had both homoeologs expressed per cluster on average across all 36 RCCs, but in individual cell types/states this value varied from around 30–70% (Figures 10A,B). Additionally, the propensity to express both WGD homoeologs as opposed to both SSD paralogs differed among cell types/states. Specifically, across all cell types/states and gene pairs, both homoeologs of alpha WGD RCC-u pairs were expressed about 1.5× more frequently than were both paralogs of tandem SSD pairs, but varied from 1.3× to nearly 2× in different RCCs (Figures 10C,D). Variable responses of different cell types/states were also seen clearly in levels of biased expression of the two paralogs of duplicate pairs in the 36 RCCs, with biased expression of alpha WGD pairs averaging around 40% across all RCCs, but varying from less than 25% to nearly 45% showing bias depending on the cell type/state (Figure 6). We attribute this variation to functional differences between cell types/states being reflected in the kinds of genes being expressed there, which in turn is connected directly to the mechanisms that lead, through expression differences, to differential retention of pairs produced by different types of duplication (Tasdighian et al., 2017; Qiao et al., 2019).

### Single Cell Data vs. Single Cell Type/State Data

Most of our results were based on root cell types and states, not on individual cells. This is a potential limitation, if it is desirable to assay gene expression at the most fundamental level both qualitatively and quantitatively. The difficulties of doing so are both technical and biological. Technically, there is a tradeoff between number of cells and number of transcripts that can be assayed; in the system used to generate these data, the “dropout” effect is a particular problem, and can distort estimates of transcript numbers, particularly between highly and weakly expressed genes (Bhargava et al., 2014; Lähnemann et al., 2020).

Biologically, it is now known that, in contrast to early concepts of gene regulation, expression is stochastic and noisy, with bursts of transcription interspersed with inactivity, and the levels of transcripts, and thus proteins, depend on complex dynamics of expression and degradation (Araujo et al., 2017; Nicholson, 2019; Tunnacliffe and Chubb, 2020). This complexity has not been incorporated into models that seek to explain duplicate gene retention and loss. As an example, for a protein encoded by a pair of paralogs and for which dosage balance maintenance is important (e.g., as part of a multi-subunit complex), it is the sum of the expression of both paralogs that is critical; the numbers of transcripts produced from either paralog is less important in the short term, but potentially dictates whether one paralog will ultimately be lost in the process of fractionation (Gout and Lynch, 2015). This revised version of the duplication-degeneration-complementation (DDC) model (Force et al., 1999) involves gradual evolution of stochastic differences in paralog expression, but it is now clear that stochastic differences are also part of the transcriptional process itself, and that these also can play a role in promoting the preservation of duplicate genes (Rodrigo and Fares, 2018; Chapal et al., 2019).

A particular scA/scB transcript ratio for a paralog pair in a cell cluster could be due to all cells expressing that ratio (rheostat-like control of expression); to an on/off mechanism, with subsets of the cell population expressing scA and scB, creating the scA/scB ratio by modulating the relative number of cells expressing each gene (Nicholson, 2019); or to more complex modes of transcriptional control (Tunnacliffe and Chubb, 2020). For polyploids, particularly allopolyploids that combine diverged genomes, there exists the possibility that a single cell cluster might include individual cells that are differentiated in their expression by subgenome. This seems unlikely, given the integration observed for higher level phenotypes – an allopolyploid individual is not a mosaic for the two different floral morphologies of its progenitors, for example, but instead integrates the developmental gene networks of its progenitors to produce a distinctive flower. However, given the idea that duplicate gene regulatory networks can diverge from one another (Blanc and Wolfe, 2004), which we see here among root cell clusters (Figure 8), it is worth looking for evidence of higher-order independence of genome expression, and the level of the cell is a natural place to look. Despite our being able to analyze only a small number of gene pair × cell combinations with statistical rigor, it appears that although most gene pairs express both paralogs in individual cells (which is most consistent with a rheostat model), there do appear to be cases where expression of paralogs is partitioned into discrete cells (Table 2). As single cell technology improves, it will be interesting to look at larger numbers of cells for any evidence of differentiation by subgenome, and to quantify the extent of deterministic (i.e., rheostat) vs. stochastic (i.e., on/off) gene regulation by determining if differences in expression among cell types are achieved by changes in the per-cell expression level or by changes in the fraction of cells expressing the gene.

### Conclusion and Future Prospects

The ability to assay transcript accumulation at the level of cell types and their developmental states permits elucidation of gene expression patterns at a very fine scale, and although the results we present here largely fall short of true single cell transcriptomics, it is almost certain that this and other current technical challenges will be overcome (Lähnemann et al., 2020). We can look forward soon to being able to compare the expression of gene pairs not only in root cells, but in all cells of the plant, eliminating the need to compare root single cell data with non-root transcriptomes generated from tissues or whole organs, as we did here.

Our characterization of the patterns of duplicate gene expression has identified a wealth of examples of differential expression of gene pairs among root cell types, the first step in achieving a detailed understanding of such issues as how pairs of homoeologs are regulated in allopolyploids at the level of trans-acting factors and cis-regulatory elements (Hu and Wendel, 2019). Single cell tools already exist for addressing these issues by assaying chromatin accessibility (Farmer et al., 2020) and mapping the epigenomic landscape of the cell (Zhou et al., 2019). For understanding functional differences between paralogs, and for even finer scale “sub-localization” of gene action within cells that may be a driver of paralog retention (Qiu et al., 2019), a host of other single cell-omics tools exist or are under development (Macaulay et al., 2017; Hasle et al., 2020).

We can learn much from single cell expression studies of a single accession of *A. thaliana*, but it is known that gene expression varies considerably among genotypes of this species (Cortijo et al., 2019), and that the response even to autopolyploidy in *Arabidopsis* varies among accessions (Yu et al., 2010; Song et al., 2020), so we look forward to the availability in expression atlases (e.g., Papatheodorou et al., 2019) of single cell data for roots and other tissues of additional individuals and accessions, and also from other species. Interspecific data would allow us to hypothesize ancestral states with far more confidence, particularly data from Cleomaceae, the sister family to Brassicaceae, whose common ancestor pre-dates the alpha WGD event (Edger et al., 2018).

Moreover, there are fascinating questions about the evolution of cell type transcriptomes that can only be addressed with comparative studies. For example, Liang et al. (2018) describe the phenomenon of “correlated evolution” of transcriptomes, in which transcriptomes of different (non-homologous) tissues of a species cluster together, instead of transcriptomes of homologous tissues in different species clustering together as expected. This is thought to be a consequence of the non-independence of transcriptomes in cell types that share transcription factors and their target genes. It is reminiscent of concerted evolution in gene families and concerted divergence of gene regulatory networks, both of which are phenomena of considerable interest and importance in polyploids (Blanc and Wolfe, 2004; Qiao et al., 2019). Comparative single cell data from other *Arabidopsis* species, including from recently formed allopolyploids in this excellent model system, hold much promise for addressing these and other questions.

## Data Availability Statement

The datasets presented in this study can be found in online repositories. The names of the repository/repositories and accession number(s) can be found in the article/Supplementary Material.

## Author Contributions

All authors conceived and wrote the manuscript. JC and AF conducted most analyses.

## Conflict of Interest

The authors declare that the research was conducted in the absence of any commercial or financial relationships that could be construed as a potential conflict of interest.

## Footnotes

1 https://github.com/adf-ncgr/singlecell_paralogue_expression_scripts

2 https://www.ebi.ac.uk/intact/

## Supplementary Material

Supplementary Figure S1 Correlation between fractions of genes expressed per RCC calculated using two different thresholds of expression (≥1 UMI and >1 cell).

Supplementary Figure S2 Correlation between genes expressed per RCC and cells per RCC.

Supplementary Figure S3 Distributions of root cell clusters expressed per gene **(A)** or gene pair **(B)** for each duplication mechanism.

Supplementary Figure S4 Bulk tissue RNA-seq based estimates of expression for Class 1 genes. **(A)** Expression breadth (number of SRA libraries out of the 214 libraries examined expressing the gene at ≥5 reads). **(B)** Expression breadth in non-root tissues (number of SRA libraries out of the 92 non-root tissue containing SRA libraries examined expressing the gene at ≥5 reads). **(C)** Expression level (combined read count from all 214 SRA libraries). **(D)** Expression level in non-root tissues (combined read count from 92 non-root tissue containing SRA libraries). “Class_1_scA” are the copies of Class 1 gene pairs expressed in the single cell root RCCs. “Class_1_scB” are the paralogs of Class 1 gene pairs not expressed in root RCCs. “Other” is the set of all other nuclear genes in *Arabidopsis* (Araport 11).

Supplementary Figure S5 RCC-u genes are enriched for WGD duplicates. Blue bars and orange bars indicate the fraction of genes that retain duplicates from the indicated mechanism for the whole genome and the RCC-u genes, respectively. The RCC-u gene set has a higher fraction of WGD duplicates and older TEs, and lower fraction of SSDs and singletons, relative to the whole genome.

Supplementary Table S1 Single cell clustering into the 36 RCCs.

Supplementary Table S2 Bulk tissue expression data.

Supplementary Table S3 Single cell expression data, properties of duplicate gene pairs, and GO enrichment data for Class 4 pairs.

Supplementary Table S4 Counts of paralogous gene pairs by expression class and duplication type.

Supplementary Table S5 Counts of scB expression for Class I paralogs.

Supplementary Table S6 K_a__/_K_s_ by expression class and duplication type.

Supplementary Table S7 Paralog expression patterns across developmental states within cell types.

Supplementary Table S8 Breakdown by expression class and duplication mechanism of 4,599 Wang et al. (2013) paralog pairs ubiquitously expressed in root cell clusters (RCC-u gene pairs).

Supplementary Table S9 Frequency of expression bias by duplication type for RCC-u and non-RCC-u pairs.

## References

[B1] Adams K. L. Cronn R. Percifield R. Wendel J. F. (2003). Genes duplicated by polyploidy show unequal contributions to the transcriptome and organ-specific reciprocal silencing. *Proc. Natl. Acad. Sci. U.S.A.* 100 4649–4654. 10.1073/pnas.0630618100 12665616 PMC153610

[B2] Araujo I. S. Pietsch J. M. Keizer E. M. Greese B. Balkunde R. Fleck C. (2017). Stochastic gene expression in *Arabidopsis thaliana*. *Nat. Commun.* 8:2132. 10.1038/s41467-017-02285-7 PMC5730595 29242599

[B3] Arendt D. Musser J. M. Baker C. V. Bergman A. Cepko C. Erwin D. H. (2016). The origin and evolution of cell types. *Nat. Rev. Genet.* 17 744–757. 27818507 10.1038/nrg.2016.127

[B4] Bhargava V. Head S. R. Ordoukhanian P. Mercola M. Subramaniam S. (2014). Technical variations in low-input RNA-seq methodologies. *Sci. Rep.* 4:3678. 10.1038/srep03678 PMC3890974 24419370

[B5] Birchler J. A. Veitia R. A. (2010). The gene balance hypothesis: implications for gene regulation, quantitative traits and evolution. *New Phytol.* 186 54–62. 10.1111/j.1469-8137.2009.03087.x 19925558 PMC2858765

[B6] Birchler J. A. Veitia R. A. (2012). Gene balance hypothesis: connecting issues of dosage sensitivity across biological disciplines. *Proc. Natl. Acad. Sci. U.S.A.* 109 14746–14753. 10.1073/pnas.1207726109 22908297 PMC3443177

[B7] Birchler J. A. Veitia R. A. (2014). The Gene Balance Hypothesis: dosage effects in plants. *Methods Mol. Biol.* 1112 25–32. 10.1007/978-1-62703-773-0_2 24478005

[B8] Birnbaum K. Shasha D. E. Wang J. Y. Jung J. W. Lambert G. M. Galbraith D. W. (2003). A gene expression map of the *Arabidopsis* root. *Science* 302 1956–1960. 10.1126/science.1090022 14671301

[B9] Blanc G. Wolfe K. H. (2004). Functional divergence of duplicated genes formed by polyploidy during *Arabidopsis* evolution. *Plant Cell* 16 1679–1691. 10.1105/tpc.021410 15208398 PMC514153

[B10] Buggs R. J. Wendel J. F. Doyle J. J. Soltis D. E. Soltis P. S. Coate J. E. (2014). The legacy of diploid progenitors in allopolyploid gene expression patterns. *Philos. Trans. R. Soc. Lond. Ser. B, Biol. Sci.* 369:20130354. 10.1098/rstb.2013.0354 24958927 PMC4071527

[B11] Butler A. Hoffman P. Smibert P. Papalexi E. Satija R. (2018). Integrating single-cell transcriptomic data across different conditions, technologies, and species. *Nat. Biotechnol.* 36 411–420. 10.1038/nbt.4096 29608179 PMC6700744

[B12] Chapal M. Mintzer S. Brodsky S. Carmi M. Barkai N. (2019). Resolving noise-control conflict by gene duplication. *PLoS Biol.* 17:e3000289. 10.1371/journal.pbio.3000289 31756183 PMC6874299

[B13] Cheng C. Y. Krishnakumar V. Chan A. P. Thibaud-Nissen F. Schobel S. Town C. D. (2017). Araport11: a complete reannotation of the *Arabidopsis thaliana* reference genome. *Plant J.* 89 789–804. 10.1111/tpj.13415 27862469

[B14] Cheng F. Wu J. Cai X. Liang J. Freeling M. Wang X. (2018). Gene retention, fractionation and subgenome differences in polyploid plants. *Nat. Plants* 4 258–268. 10.1038/s41477-018-0136-7 29725103

[B15] Coate J. E. Song M. J. Bombarely A. Doyle J. J. (2016). Expression-level support for gene dosage sensitivity in three *Glycine* subgenus *Glycine* polyploids and their diploid progenitors. *New Phytol.* 212 1083–1093. 10.1111/nph.14090 27418296

[B16] Cortijo S. Aydin Z. Ahnert S. Locke J. C. (2019). Widespread inter-individual gene expression variability in *Arabidopsis thaliana*. *Mol. Syst. Biol.* 15:e8591. 10.15252/msb.20188591 PMC6346214 30679203

[B17] Defoort J. Van de Peer Y. Carretero-Paulet L. (2019). The Evolution of Gene Duplicates in Angiosperms and the Impact of Protein-Protein Interactions and the Mechanism of Duplication. *Genome Biol. Evol.* 11 2292–2305. 31364708 10.1093/gbe/evz156 PMC6735927

[B18] Denyer T. Ma X. Klesen S. Scacchi E. Nieselt K. Timmermans M. C. P. (2019). Spatiotemporal Developmental Trajectories in the *Arabidopsis* Root Revealed Using High-Throughput Single-Cell RNA Sequencing. *Dev. Cell.* 48 840–852.e845. 30913408 10.1016/j.devcel.2019.02.022

[B19] Edger P. P. Hall J. C. Harkess A. Tang M. Coombs J. Mohammadin S. (2018). Brassicales phylogeny inferred from 72 plastid genes: a reanalysis of the phylogenetic localization of two paleopolyploid events and origin of novel chemical defenses. *Am. J. Bot.* 105 463–469. 10.1002/ajb2.1040 29574686

[B20] Efroni I. Birnbaum K. D. (2016). The potential of single-cell profiling in plants. *Genome Biol.* 17 1–8. 10.1002/9783527678679.dg11957 27048384 PMC4820866

[B21] Emery M. Willis M. M. S. Hao Y. Barry K. Oakgrove K. Peng Y. (2018). Preferential retention of genes from one parental genome after polyploidy illustrates the nature and scope of the genomic conflicts induced by hybridization. *PLoS Genet.* 14:e1007267. 10.1371/journal.pgen.1007267 29590103 PMC5891031

[B22] Farmer A. Thibivilliers S. Ryu K. H. Schiefelbein J. Libault M. (2020). The impact of chromatin remodeling on gene expression at the single cell level in *Arabidopsis thaliana*. *bioRxiv [Preprint]* 10.1101/2020.07.27.223156 33422696

[B23] Force A. Lynch M. Pickett F. B. Amores A. Yan Y. L. Postlethwait J. (1999). Preservation of duplicate genes by complementary, degenerative mutations. *Genetics* 151 1531–1545. 10101175 10.1093/genetics/151.4.1531 PMC1460548

[B24] Freeling M. (2009). Bias in plant gene content following different sorts of duplication: tandem, whole-genome, segmental, or by transposition. *Annu. Rev. Plant Biol.* 60 433–453. 10.1146/annurev.arplant.043008.092122 19575588

[B25] Gabaldon T. Koonin E. V. (2013). Functional and evolutionary implications of gene orthology. *Nat. Rev. Genet.* 14 360–366. 10.1038/nrg3456 23552219 PMC5877793

[B26] Gallagher J. P. Grover C. E. Hu G. Jareczek J. J. Wendel J. F. (2020). Conservation and divergence in duplicated fiber coexpression networks accompanying domestication of the polyploid *Gossypium hirsutum* L. *G3* 10 2879–2892. 10.1534/g3.120.401362 32586849 PMC7407458

[B27] Gou J. Y. Wang L. J. Chen S. P. Hu W. L. Chen X. Y. (2007). Gene expression and metabolite profiles of cotton fiber during cell elongation and secondary cell wall synthesis. *Cell Res.* 17 422–434. 10.1038/sj.cr.7310150 17387330

[B28] Gout J. F. Lynch M. (2015). Maintenance and loss of duplicated genes by dosage subfunctionalization. *Mol. Biol. Evol.* 32 2141–2148. 10.1093/molbev/msv095 25908670 PMC4833079

[B29] Grover C. E. Gallagher J. P. Szadkowski E. P. Yoo M. J. Flagel L. E. Wendel J. F. (2012). Homoeolog expression bias and expression level dominance in allopolyploids. *New Phytol.* 196 966–971. 10.1111/j.1469-8137.2012.04365.x 23033870

[B30] Hanada K. Kuromori T. Myouga F. Toyoda T. Shinozaki K. (2009). Increased expression and protein divergence in duplicate genes is associated with morphological diversification. *PLoS Genet.* 5:e1000781. 10.1371/journal.pgen.1000781 20041196 PMC2788128

[B31] Hao Y. Washburn J. D. Rosenthal J. Nielsen B. Lyons E. Edger P. P. (2018). Patterns of population variation in two paleopolyploid eudicot lineages suggest that dosage-based selection on homeologs is long-lived. *Genome Biol. Evol.* 10 999–1011. 10.1093/gbe/evy061 29617811 PMC5887293

[B32] Hasle N. Cooke A. Srivatsan S. Huang H. Stephany J. J. Krieger Z. (2020). High-throughput, microscope-based sorting to dissect cellular heterogeneity. *Mol. Syst. Biol.* 16:e9442. 10.15252/msb.20209442 PMC7273721 32500953

[B33] Honys D. Twell D. (2004). Transcriptome analysis of haploid male gametophyte development in *Arabidopsis*. *Genome Biol.* 5:13. 10.1186/gb-2004-5-11-r85 PMC545776 15535861

[B34] Hossain M. S. Joshi T. Stacey G. (2015). System approaches to study root hairs as a single cell plant model: current status and future perspectives. *Front. Plant Sci.* 6:363. 10.3389/fpls.2015.00363 26042143 PMC4436566

[B35] Hovav R. Udall J. A. Chaudhary B. Rapp R. Flagel L. Wendel J. F. (2008a). Partitioned expression of duplicated genes during development and evolution of a single cell in a polyploid plant. *Proc. Natl. Acad. Sci. U.S.A.* 105 6191–6195. 10.1073/pnas.0711569105 18420816 PMC2329682

[B36] Hovav R. Udall J. A. Hovav E. Rapp R. Flagel L. Wendel J. F. (2008b). A majority of cotton genes are expressed in single-celled fiber. *Planta* 227 319–329. 10.1007/s00425-007-0619-7 17849148

[B37] Hu G. Wendel J. F. (2019). Cis-trans controls and regulatory novelty accompanying allopolyploidization. *New Phytol.* 221 1691–1700. 10.1111/nph.15515 30290011

[B38] Huang L. Schiefelbein J. (2015). Conserved Gene Expression Programs in Developing Roots from Diverse Plants. *Plant Cell* 27 2119–2132. 10.1105/tpc.15.00328 26265761 PMC4568505

[B39] Innan H. Kondrashov F. (2010). The evolution of gene duplications: classifying and distinguishing between models. *Nat. Rev. Genet.* 11 97–108. 10.1038/nrg2689 20051986

[B40] Jean-Baptiste K. McFaline-Figueroa J. L. Alexandre C. M. Dorrity M. W. Saunders L. Bubb K. L. (2019). Dynamics of gene expression in single root cells of *Arabidopsis thaliana*. *Plant Cell* 31 993–1011. 10.1105/tpc.18.00785 30923229 PMC8516002

[B41] Jiao Y. Leebens-Mack J. Ayyampalayam S. Bowers J. E. McKain M. R. McNeal J. (2012). A genome triplication associated with early diversification of the core eudicots. *Genome Biol.* 13:R3. 10.1186/gb-2012-13-1-r3 PMC3334584 22280555

[B42] Kenrick P. Strullu-Derrien C. (2014). The origin and early evolution of roots. *Plant Physiol.* 166 570–580. 10.1104/pp.114.244517 25187527 PMC4213089

[B43] Kim D. Paggi J. M. Park C. Bennett C. Salzberg S. L. (2019). Graph-based genome alignment and genotyping with HISAT2 and HISAT-genotype. *Nat. Biotechnol.* 37 907–915. 10.1038/s41587-019-0201-4 31375807 PMC7605509

[B44] Kovaka S. Zimin A. V. Pertea G. M. Razaghi R. Salzberg S. L. Pertea M. (2019). Transcriptome assembly from long-read RNA-seq alignments with StringTie2. *Genome Biol.* 20:278. 10.1186/s13059-019-1910-1 PMC6912988 31842956

[B45] Lähnemann D. Köster J. Szczurek E. McCarthy D. J. Hicks S. C. Robinson M. D. (2020). Eleven grand challenges in single-cell data science. *Genome Biol.* 21:31. 10.1186/s13059-020-1926-6 PMC7007675 32033589

[B46] Langham R. J. Walsh J. Dunn M. Ko C. Goff S. A. Freeling M. (2004). Genomic duplication, fractionation and the origin of regulatory novelty. *Genetics* 166 935–945. 10.1534/genetics.166.2.935 15020478 PMC1470742

[B47] Leebens-Mack J. H. Barker M. S. Carpenter E. J. Deyholos M. K. Gitzendanner M. A. Graham S. W. (2019). One thousand plant transcriptomes and the phylogenomics of green plants. *Nature* 574 679–685. 10.1038/s41586-019-1693-2 31645766 PMC6872490

[B48] Leinonen R. Sugawara H. Shumway M. (2011). International Nucleotide Sequence Database Collaboration. The sequence read archive. *Nucleic Acids Res.* 39 D19–D21. 10.1093/nar/gkq1019 21062823 PMC3013647

[B49] Liang C. Musser J. M. Cloutier A. Prum R. O. Wagner G. P. (2018). Pervasive correlated evolution in gene expression shapes cell and tissue type transcriptomes. *Genome Biol. Evol.* 10 538–552. 10.1093/gbe/evy016 29373668 PMC5800078

[B50] Liang Z. Schnable J. C. (2018). Functional divergence between subgenomes and gene pairs after whole genome duplications. *Mol. Plant* 11 388–397. 10.1016/j.molp.2017.12.010 29275166

[B51] Libault M. Pingault L. Zogli P. Schiefelbein J. (2017). Plant systems biology at the single-cell level. *Trends Plant Sci.* 22 949–960. 10.1016/j.tplants.2017.08.006 28970001

[B52] Lloyd J. P. Tsai Z. T. Sowers R. P. Panchy N. L. Shiu S. H. (2018). A Model-Based Approach for Identifying Functional Intergenic Transcribed Regions and Noncoding RNAs. *Mol. Biol. Evol.* 35 1422–1436. 10.1093/molbev/msy035 29554332

[B53] Luecken M. D. Theis F. J. (2019). Current best practices in single-cell RNA-seq analysis: a tutorial. *Mol. Syst. Biol.* 15:e8746. 10.15252/msb.20188746 PMC6582955 31217225

[B54] Lynch M. (2020). The evolutionary scaling of cellular traits imposed by the drift barrier. *Proc. Natl. Acad. Sci. U.S.A.* 117 10435–10444. 10.1073/pnas.2000446117 32345718 PMC7229682

[B55] Lynch M. Conery J. S. (2000). The evolutionary fate and consequences of duplicate genes. *Science* 290 1151–1155. 10.1126/science.290.5494.1151 11073452

[B56] Lynch M. O’Hely M. Walsh B. Force A. (2001). The probability of preservation of a newly arisen gene duplicate. *Genetics* 159 1789–1804. 11779815 10.1093/genetics/159.4.1789 PMC1461922

[B57] Lynch M. Trickovic B. (2020). A theoretical framework for evolutionary cell biology. *J. Mol. Biol.* 432 1861–1879. 10.1016/j.jmb.2020.02.006 32087200 PMC8025685

[B58] Macaulay I. C. Ponting C. P. Voet T. (2017). Single-cell multiomics: multiple measurements from single cells. *Trends Genet.* 33 155–168. 10.1016/j.tig.2016.12.003 28089370 PMC5303816

[B59] McClintock B. (1984). The Significance of Responses of the Genome to Challenge. *Science* 226 792–801. 10.1126/science.15739260 15739260

[B60] Morris S. A. (2019). The evolving concept of cell identity in the single cell era. *Development* 146:dev169748. 10.1242/dev.169748 31249002

[B61] Nei M. Gojobori T. (1986). Simple methods for estimating the numbers of synonymous and nonsynonymous nucleotide substitutions. *Mol. Biol. Evol.* 3 418–426. 3444411 10.1093/oxfordjournals.molbev.a040410

[B62] Nicholson D. J. (2019). Is the cell really a machine? *J. Theor. Biol.* 477 108–126. 10.1016/j.jtbi.2019.06.002 31173758

[B63] Ohno S. (1970). *Evolution by Gene Duplication.* Berlin: Springer.

[B64] Panchy N. Lehti-Shiu M. Shiu S. H. (2016). Evolution of gene duplication in plants. *Plant Physiol.* 171 2294–2316. 27288366 10.1104/pp.16.00523 PMC4972278

[B65] Panchy N. L. Azodi C. B. Winship E. F. O’Malley R. C. Shiu S. H. (2019). Expression and regulatory asymmetry of retained *Arabidopsis thaliana* transcription factor genes derived from whole genome duplication. *BMC Evol. Biol.* 19:77. 10.1186/s12862-019-1398-z 30866803 PMC6416927

[B66] Papatheodorou I. Moreno P. Manning J. Fuentes A. M.-P. George N. Fexova S. (2019). Expression Atlas update: from tissues to single cells. *Nucleic Acids Res.* 48 D77–D83. 10.1093/nar/gkz947 PMC7145605 31665515

[B67] Papp B. Pál C. Hurst L. D. (2003). Dosage sensitivity and the evolution of gene families in yeast. *Nature* 424 194–197. 10.1038/nature01771 12853957

[B68] Qiao X. Li Q. Yin H. Qi K. Li L. Wang R. (2019). Gene duplication and evolution in recurring polyploidization-diploidization cycles in plants. *Genome Biol.* 20:38. 10.1186/s13059-019-1650-2 PMC6383267 30791939

[B69] Qiao Z. Libault M. (2013). Unleashing the potential of the root hair cell as a single plant cell type model in root systems biology. *Front. Plant Sci.* 4:484. 10.3389/fpls.2013.00484 24324480 PMC3840615

[B70] Qiu Y. Tay Y. V. Ruan Y. Adams K. L. (2019). Divergence of duplicated genes by repeated partitioning of splice forms and subcellular localization. *New Phytol.* 225 1011–1022. 10.1111/nph.16148 31469915

[B71] Raven J. A. Edwards D. (2001). Roots: evolutionary origins and biogeochemical significance. *J. Exp. Bot.* 52 381–401. 10.1093/jxb/52.suppl_1.381 11326045

[B72] Renny-Byfield S. Gallagher J. P. Grover C. E. Szadkowski E. Page J. T. Udall J. A. (2014). Ancient gene duplicates in Gossypium (cotton) exhibit near-complete expression divergence. *Genome Biol. Evol.* 6 559–571. 10.1093/gbe/evu037 24558256 PMC3971588

[B73] Rodrigo G. Fares M. A. (2018). Intrinsic adaptive value and early fate of gene duplication revealed by a bottom-up approach. *eLife* 7:e29739. 10.7554/eLife.29739 PMC5771667 29303479

[B74] Ryu K. H. Huang L. Kang H. M. Schiefelbein J. (2019). Single-Cell RNA Sequencing Resolves Molecular Relationships Among Individual Plant Cells. *Plant Physiol.* 179 1444–1456. 10.1104/pp.18.01482 30718350 PMC6446759

[B75] Schnable J. C. Wang X. Pires J. C. Freeling M. (2012). Escape from preferential retention following repeated whole genome duplications in plants. *Front. Plant Sci.* 3:94. 10.3389/fpls.2012.00094 22639677 PMC3355610

[B76] Shen L. Sinai M. (2019). *GeneOverlap**: Test and Visualize Gene Overlaps. R Package Version 1.23.0.* Available at: http://shenlab-sinai.github.io/shenlab-sinai/. 10.18129/B9.bioc.GeneOverlap (accessed June 3, 2020).

[B77] Shi Y. H. Zhu S. W. Mao X. Z. Feng J. X. Qin Y. M. Zhang L. (2006). Transcriptome profiling, molecular biological, and physiological studies reveal a major role for ethylene in cotton fiber cell elongation. *Plant Cell.* 18 651–664. 10.1105/tpc.105.040303 16461577 PMC1383640

[B78] Shulse C. N. Cole B. J. Ciobanu D. Lin J. Yoshinaga Y. Gouran M. (2019). High-throughput single-cell transcriptome profiling of plant cell types. *Cell Rep.* 27 2241–2247.e2244. 31091459 10.1016/j.celrep.2019.04.054 PMC6758921

[B79] Song M. J. Potter B. I. Doyle J. J. Coate J. E. (2020). Gene balance predicts transcriptional responses immediately following ploidy change in *Arabidopsis thaliana*. *Plant Cell* 32 1434–1448. 10.1105/tpc.19.00832 32184347 PMC7203931

[B80] Stajich J. E. Block D. Boulez K. Brenner S. E. Chervitz S. A. Dagdigian C. (2002). The Bioperl toolkit: Perl modules for the life sciences. *Genome Res.* 12 1611–1618. 10.1101/gr.361602 12368254 PMC187536

[B81] Steige K. A. Slotte T. (2016). Genomic legacies of the progenitors and the evolutionary consequences of allopolyploidy. *Curr. Opin. Plant Biol.* 30 88–93. 10.1016/j.pbi.2016.02.006 26943938

[B82] Taliercio E. W. Boykin D. (2007). Analysis of gene expression in cotton fiber initials. *BMC Plant Biol.* 7:22. 10.1186/1471-2229-7-22 17506877 PMC1906782

[B83] Tasdighian S. Van Bel M. Li Z. Van de Peer Y. Carretero-Paulet L. Maere S. (2017). Reciprocally retained genes in the angiosperm lineage show the hallmarks of dosage balance sensitivity. *Plant Cell* 29 2766–2785. 10.1105/tpc.17.00313 29061868 PMC5728127

[B84] Tunnacliffe E. Chubb J. R. (2020). What is a transcriptional burst? *Trends Genet.* 36 288–297. 10.1016/j.tig.2020.01.003 32035656

[B85] Van de Peer Y. Mizrachi E. Marchal K. (2017). The evolutionary significance of polyploidy. *Nat. Rev. Genet.* 18 411–424. 10.1038/nrg.2017.26 28502977

[B86] Vickaryous M. K. Hall B. K. (2006). Human cell type diversity, evolution, development, and classification with special reference to cells derived from the neural crest. *Biol. Rev. Cambridge Philos. Soc.* 81 425–455. 10.1017/s1464793106007068 16790079

[B87] Wang Y. Tan X. Paterson A. H. (2013). Different patterns of gene structure divergence following gene duplication in Arabidopsis. *BMC Genomics* 14:652. 10.1186/1471-2164-14-652 24063813 PMC3848917

[B88] Wang Y. Tang H. Debarry J. D. Tan X. Li J. Wang X. (2012). MCScanX: a toolkit for detection and evolutionary analysis of gene synteny and collinearity. *Nucleic Acids Res.* 40 e49. 10.1093/nar/gkr1293 22217600 PMC3326336

[B89] Wendel J. F. (2015). The wondrous cycles of polyploidy in plants. *Am. J. Bot.* 102 1753–1756. 10.3732/ajb.1500320 26451037

[B90] Wilson E. B. (1927). Probable inference, the law of succession, and statistical inference. *J. Am. Stat. Assoc.* 22 209–212. 10.1080/01621459.1927.10502953

[B91] Woodhouse M. R. Cheng F. Pires J. C. Lisch D. Freeling M. Wang X. (2014). Origin, inheritance, and gene regulatory consequences of genome dominance in polyploids. *Proc. Natl. Acad. Sci. U.S.A.* 111:201402475. 10.1073/pnas.1402475111 PMC3986174 24706847

[B92] Xie J. Li Y. Liu X. Zhao Y. Li B. Ingvarsson P. K. (2019). Evolutionary origins of pseudogenes and their association with regulatory sequences in plants. *Plant Cell* 31 563–578. 10.1105/tpc.18.00601 30760562 PMC6482637

[B93] Yoo M. J. Szadkowski E. Wendel J. F. (2013). Homoeolog expression bias and expression level dominance in allopolyploid cotton. *Heredity* 110 171–180. 10.1038/hdy.2012.94 23169565 PMC3554454

[B94] Yoo M. J. Wendel J. F. (2014). Comparative evolutionary and developmental dynamics of the cotton (*Gossypium hirsutum*) fiber transcriptome. *PLoS Genet.* 10:e1004073. 10.1371/journal.pgen.1004073 24391525 PMC3879233

[B95] Yu Z. Haberer G. Matthes M. Rattei T. Mayer K. F. Gierl A. (2010). Impact of natural genetic variation on the transcriptome of autotetraploid *Arabidopsis thaliana*. *Proc. Natl. Acad. Sci. U.S.A.* 107 17809–17814. 20876110 10.1073/pnas.1000852107 PMC2955106

[B96] Yuan M. Yang X. Lin J. Cao X. Chen F. Zhang X. (2020). Alignment of cell lineage trees elucidates genetic programs for the development and evolution of cell types. *iScience* 23 101273–101273. 10.1016/j.isci.2020.101273 32599560 PMC7327887

[B97] Zhang T. Q. Xu Z. G. Shang G. D. Wang J. W. (2019). A Single-Cell RNA sequencing profiles the developmental landscape of arabidopsis root. *Mol. Plant* 12 648–660. 10.1016/j.molp.2019.04.004 31004836

[B98] Zhou S. Jiang W. Zhao Y. Zhou D. X. (2019). Single-cell three-dimensional genome structures of rice gametes and unicellular zygotes. *Nat. Plants* 5 795–800. 10.1038/s41477-019-0471-3 31332313

