# Supplementary material for: Expression Partitioning of Duplicate Genes at Single Cell Resolution in Arabidopsis Roots
Source: Front Genet. 2020 Nov 3;11:596150. doi: 10.3389/fgene.2020.596150 (PMC7670048; doi:10.3389/fgene.2020.596150)
Supplement: Supplementary Figure S1 — Correlation between fractions of genes expressed per RCC calculated using two different thresholds of expression (≥1 UMI and >1 cell). [file Presentation_1.PPTX]

## Slide 1
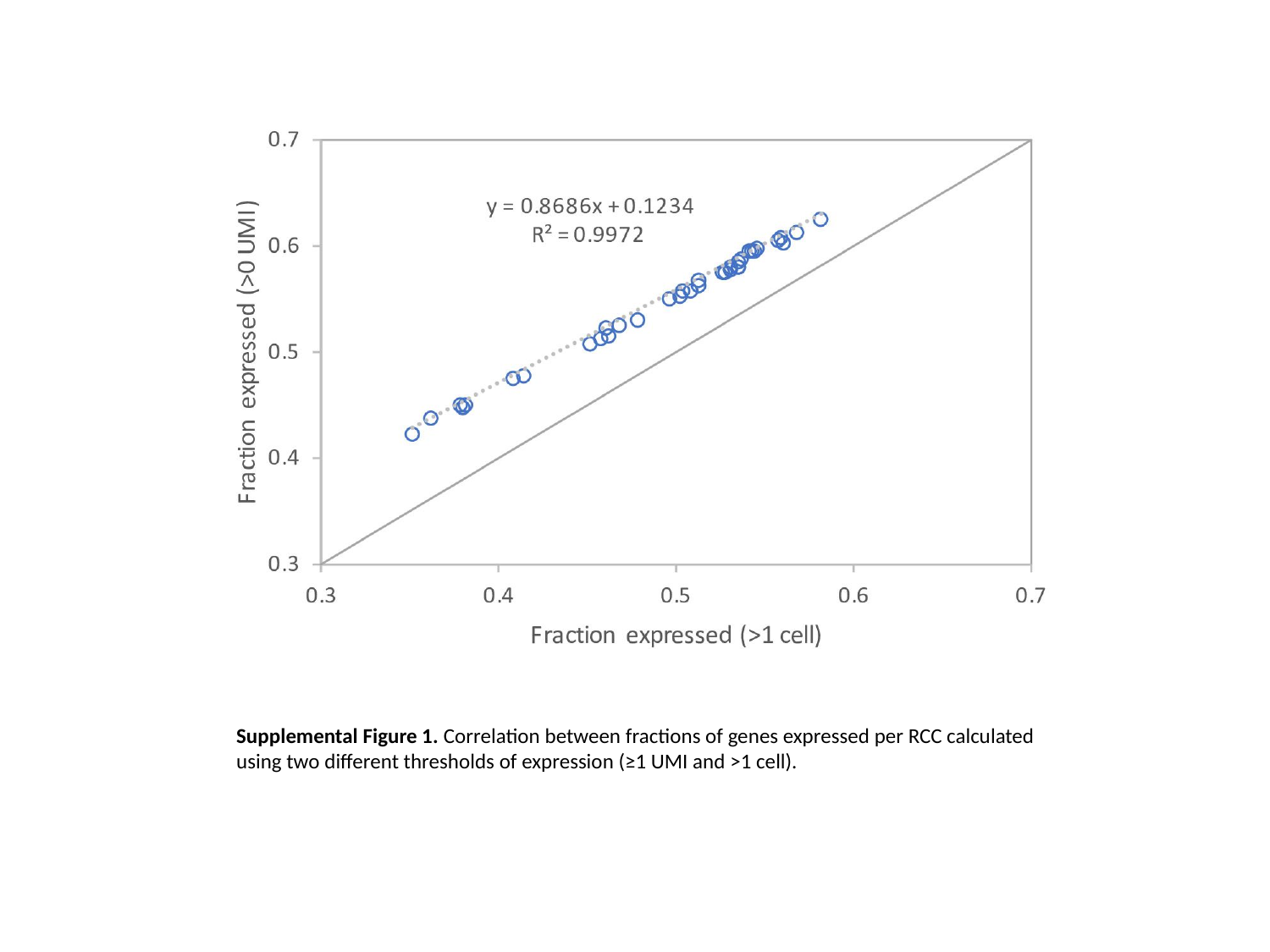

Supplemental Figure 1. Correlation between fractions of genes expressed per RCC calculated using two different thresholds of expression (≥1 UMI and >1 cell).

## Slide 2
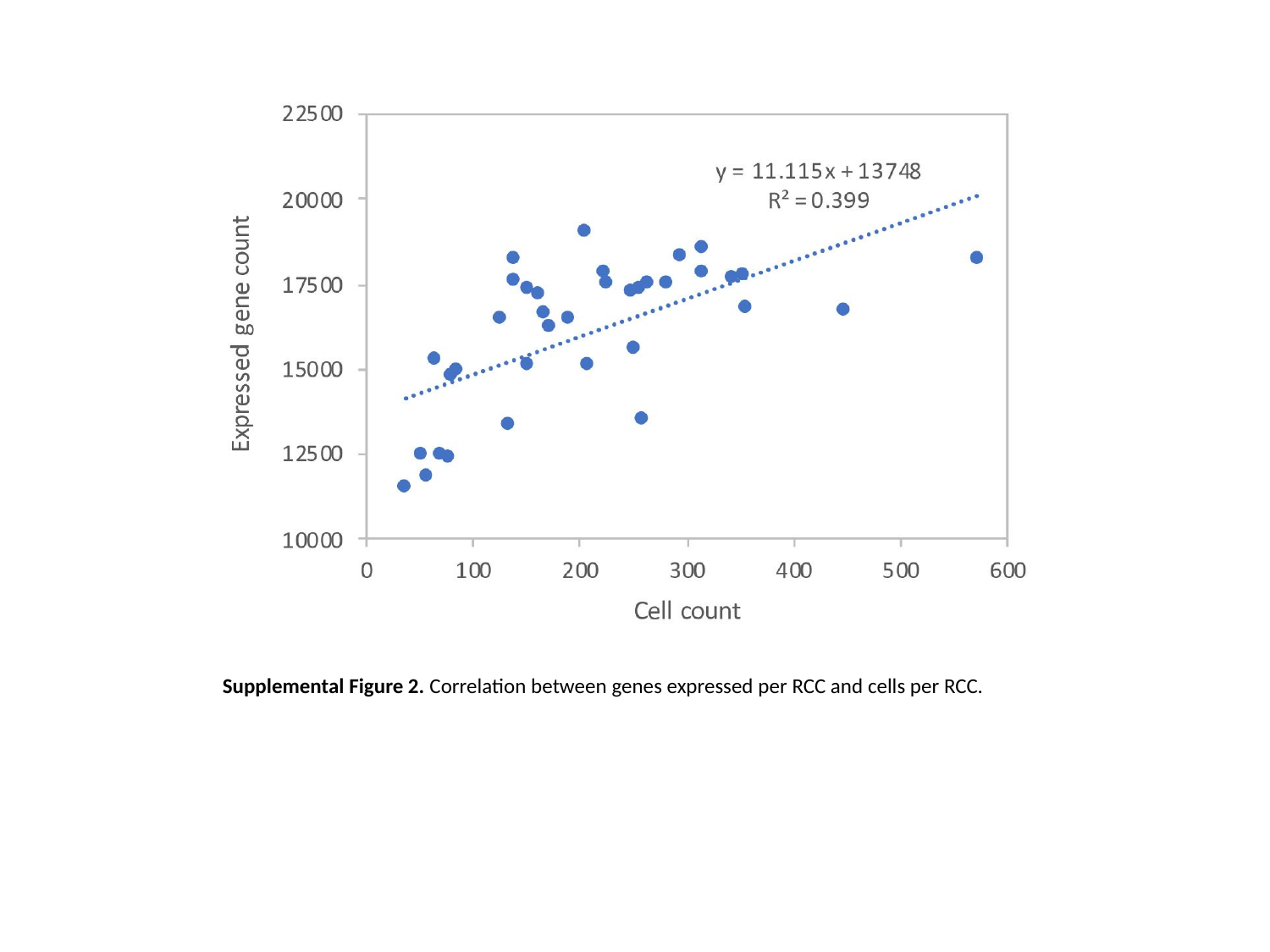

Supplemental Figure 2. Correlation between genes expressed per RCC and cells per RCC.

## Slide 3
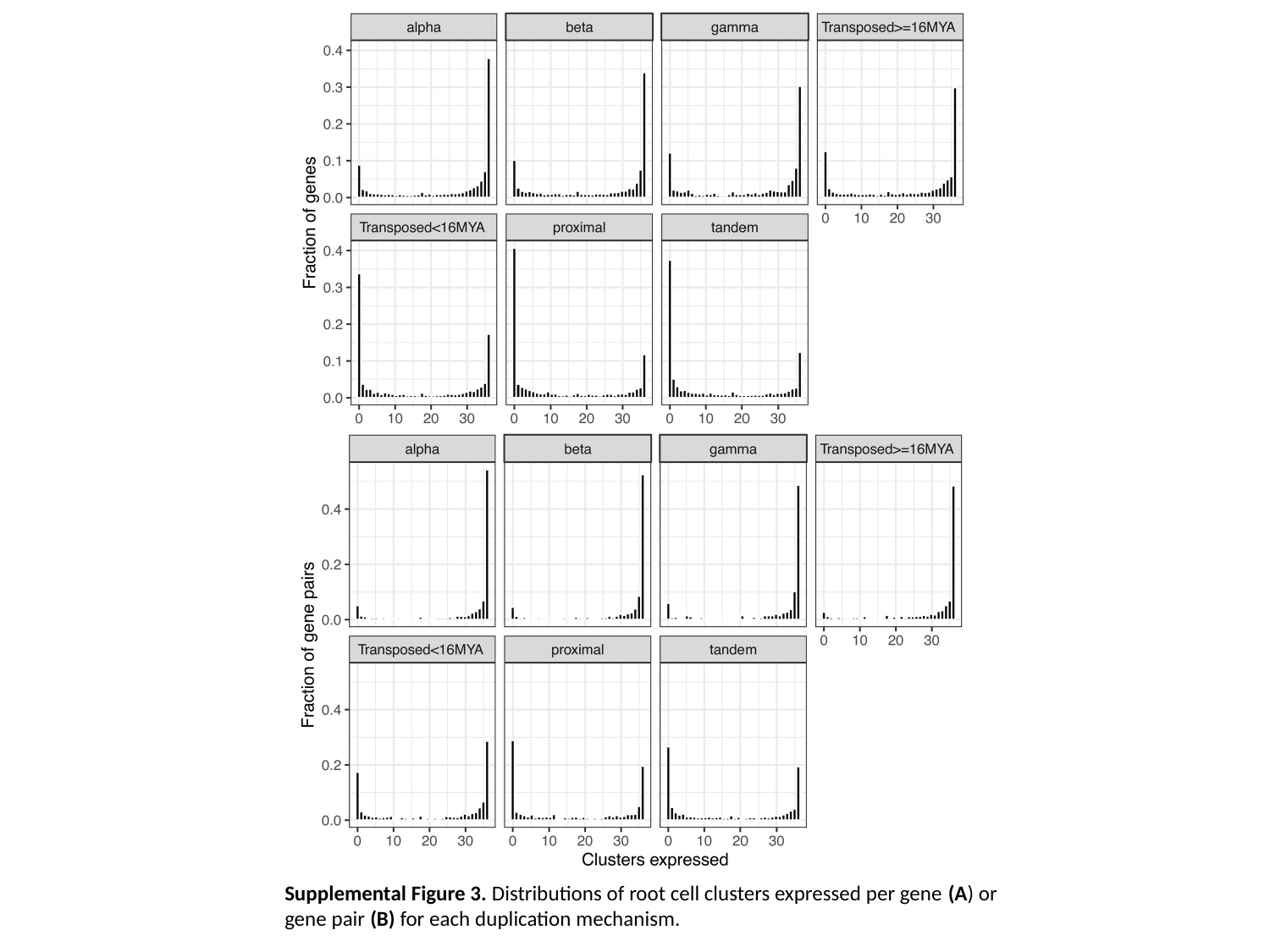

Supplemental Figure 3. Distributions of root cell clusters expressed per gene (A) or gene pair (B) for each duplication mechanism.

## Slide 4
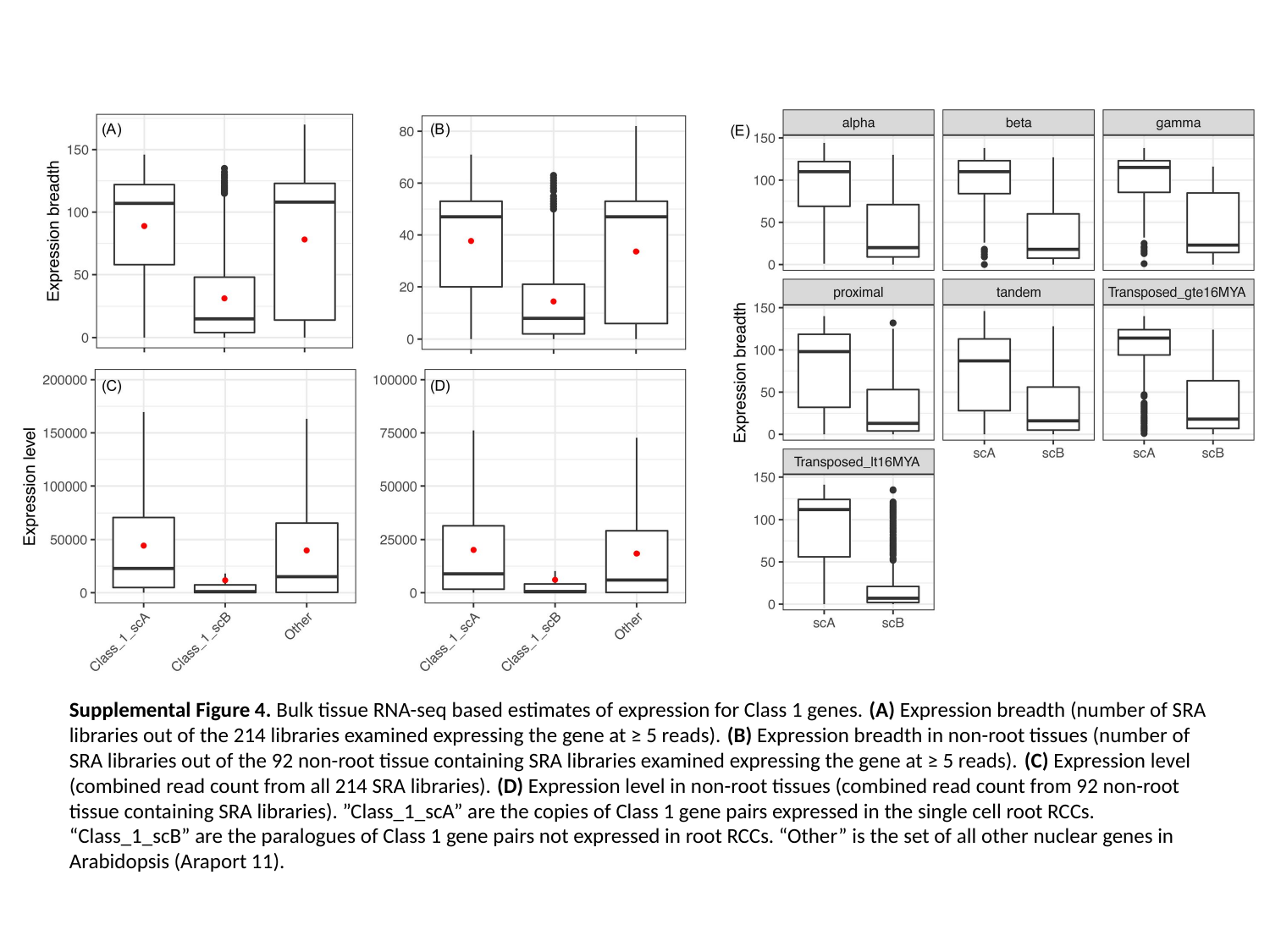

Supplemental Figure 4. Bulk tissue RNA-seq based estimates of expression for Class 1 genes. (A) Expression breadth (number of SRA libraries out of the 214 libraries examined expressing the gene at ≥ 5 reads). (B) Expression breadth in non-root tissues (number of SRA libraries out of the 92 non-root tissue containing SRA libraries examined expressing the gene at ≥ 5 reads). (C) Expression level (combined read count from all 214 SRA libraries). (D) Expression level in non-root tissues (combined read count from 92 non-root tissue containing SRA libraries). ”Class_1_scA” are the copies of Class 1 gene pairs expressed in the single cell root RCCs. “Class_1_scB” are the paralogues of Class 1 gene pairs not expressed in root RCCs. “Other” is the set of all other nuclear genes in Arabidopsis (Araport 11).

## Slide 5
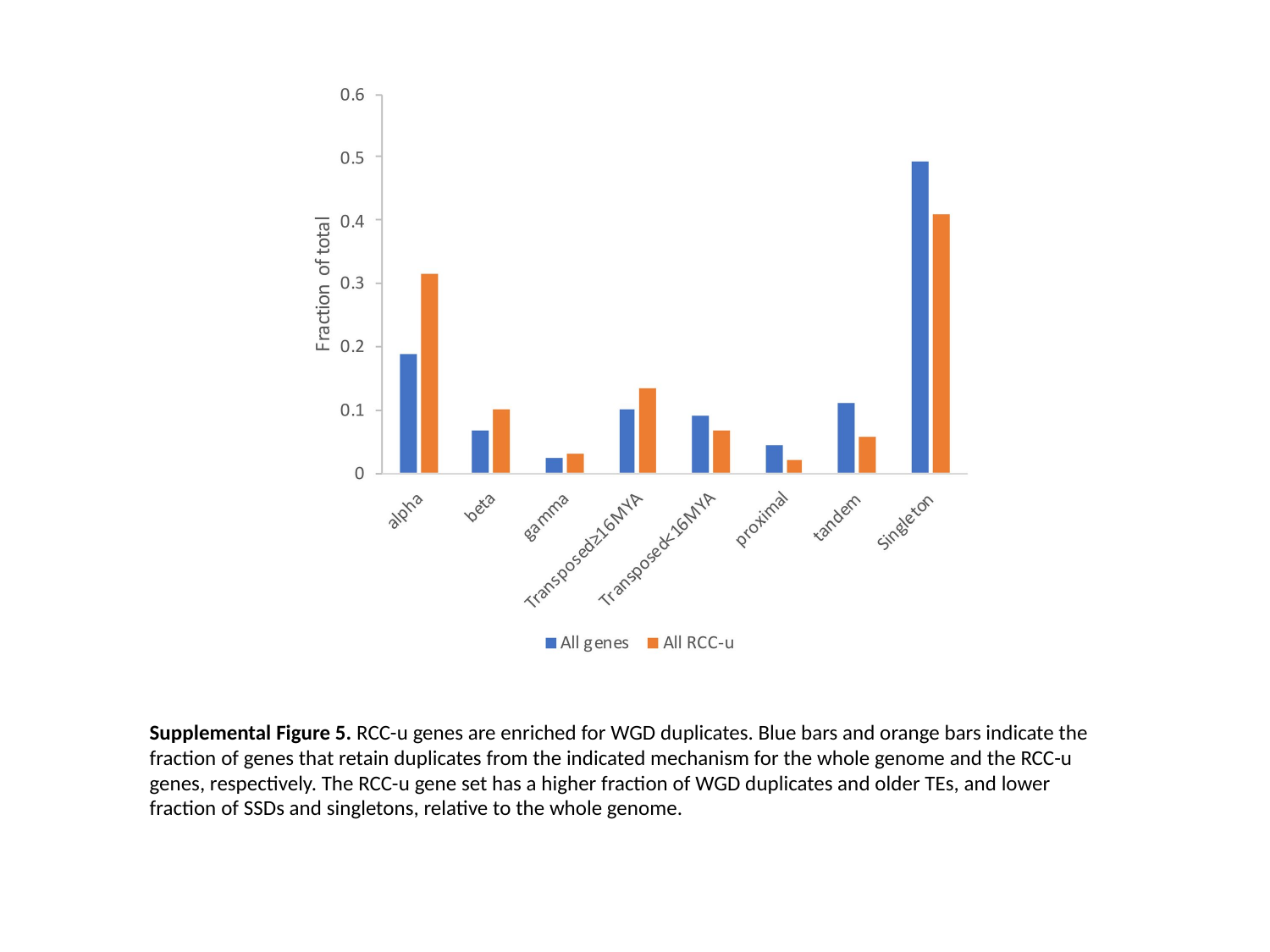

Supplemental Figure 5. RCC-u genes are enriched for WGD duplicates. Blue bars and orange bars indicate the fraction of genes that retain duplicates from the indicated mechanism for the whole genome and the RCC-u genes, respectively. The RCC-u gene set has a higher fraction of WGD duplicates and older TEs, and lower fraction of SSDs and singletons, relative to the whole genome.
