# Supplementary material for: Expression Partitioning of Duplicate Genes at Single Cell Resolution in Arabidopsis Roots
Source: Front Genet. 2020 Nov 3;11:596150. doi: 10.3389/fgene.2020.596150 (PMC7670048; doi:10.3389/fgene.2020.596150)
Supplement: Supplementary Table S4 — Counts of paralogous gene pairs by expression class and duplication type. [file Table_4.docx]

**Supplemental Table 4.** Counts of paralogous gene pairs by expression class and duplication type.

| Class | alpha | beta | gamma | Transposed ≥16MYA | Transposed  <16MYA | proximal | tandem | Total |
| --- | --- | --- | --- | --- | --- | --- | --- | --- |
| 0 | 84  (2.7%) | 37  (2.6%) | 16  (3.2%) | 52  (2.8%) | 297  (17.5%) | 223  (28.8%) | 574  (27.1%) | 1,283  (11.2%) |
| 1 | 397  (12.8%) | 219  (15.4%) | 90  (17.8%) | 363  (19.5%) | 601  (35.5%) | 223  (28.8%) | 530  (25.0%) | 2,423  (21.1%) |
| 2 | 957  (30.9%) | 265  (18.7%) | 94  (18.6%) | 508  (27.3%) | 295  (17.4%) | 122  (15.8%) | 338  (15.9%) | 2,579  (22.5%) |
| 3 | 1570  (50.7%) | 814  (57.3%) | 283  (56.0%) | 860  (46.2%) | 473  (27.9%) | 192  (24.8%) | 641  (30.2%) | 4,833  (42.1%) |
| 4 | 88  (2.8%) | 85  (6.0%) | 22  (4.4%) | 78  (4.2%) | 27  (1.6%) | 14  (1.8%) | 38  (1.8%) | 352  (3.1%) |
| Total | 3096 | 1420 | 505 | 1861 | 1693 | 774 | 2121 | 11470 |
