# Supplementary material for: Expression Partitioning of Duplicate Genes at Single Cell Resolution in Arabidopsis Roots
Source: Front Genet. 2020 Nov 3;11:596150. doi: 10.3389/fgene.2020.596150 (PMC7670048; doi:10.3389/fgene.2020.596150)
Supplement: Supplementary Table S5 — Counts of scB expression for Class I paralogs. [file Table_5.docx]

**Supplemental Table 5.** Counts of scB expression for Class I paralogues. A total of 2,423 pairs were assigned to Class 1 (scA expressed in > 1 cell/cluster in at least one cluster, scB expressed in ≤ 1 cell/cluster in all 36 RCCs).

|  |  | scB expressed at ≥5 reads in at least one SRA library | |
| --- | --- | --- | --- |
| scA expression cutoff^1^ | Class 1 pairs | All SRA libraries | Excluding root libraries^2^ |
| > 1 cell | 2,423 | 2,253 (93.0%) | 2,214 (91.4%) |
| > 1 cell, >1 RCC | 2,153 | 1,998 (92.8%) | 1,966 (91.3%) |
| > 1 cell, >5 RCC | 1,734 | 1,604 (92.5%) | 1,574 (90.8%) |

^1^Criteria for scA to be considered expressed in the root scRNA-seq libraries.

^2^121 out of 213 SRA libraries whose metadata indicate they were derived from root tissue or included root tissue (e.g., whole seedlings), were excluded from the analysis.
