# Supplementary material for: Expression Partitioning of Duplicate Genes at Single Cell Resolution in Arabidopsis Roots
Source: Front Genet. 2020 Nov 3;11:596150. doi: 10.3389/fgene.2020.596150 (PMC7670048; doi:10.3389/fgene.2020.596150)
Supplement: Supplementary Table S6 — Ka/Ks by expression class and duplication type. [file Table_6.docx]

**Supplemental Table 6.** K_a/_K_s_ by expression Class and duplication type.

|  |  |  | Ka/Ks | |
| --- | --- | --- | --- | --- |
| Class | Duplication type | Count | Mean | SD |
| 0 | all | 1,283 | 0.79 | 1.06 |
|  | alpha | 84 | 0.30 | 0.18 |
|  | beta | 37 | 0.59 | 0.69 |
|  | gamma | 16 | 0.46 | 0.26 |
|  | Transposed≥16 MYA | 52 | 0.43 | 0.34 |
|  | Transposed<16 MYA | 297 | 0.96 | 1.58 |
|  | proximal | 223 | 1.01 | 1.39 |
|  | tandem | 574 | 0.75 | 0.61 |
| 1 | all | 2,423 | 0.70 | 1.19 |
|  | alpha | 397 | 0.39 | 0.45 |
|  | beta | 219 | 0.44 | 0.64 |
|  | gamma | 90 | 0.41 | 0.65 |
|  | Transposed≥16 MYA | 363 | 0.52 | 0.52 |
|  | Transposed<16 MYA | 601 | 1.00 | 1.54 |
|  | proximal | 223 | 0.87 | 0.96 |
|  | tandem | 530 | 0.79 | 1.60 |
| 2 | all | 2,577 | 0.44 | 0.95 |
|  | alpha | 957 | 0.22 | 0.19 |
|  | beta | 263 | 0.30 | 0.33 |
|  | gamma | 94 | 0.45 | 0.61 |
|  | Transposed≥16 MYA | 508 | 0.61 | 0.59 |
|  | Transposed<16 MYA | 295 | 0.66 | 0.82 |
|  | proximal | 122 | 0.96 | 3.67 |
|  | tandem | 338 | 0.52 | 0.56 |
| 3 | all | 4,835 | 0.41 | 0.68 |
|  | alpha | 1,570 | 0.27 | 0.32 |
|  | beta | 816 | 0.31 | 0.47 |
|  | gamma | 283 | 0.39 | 0.52 |
|  | Transposed≥16 MYA | 860 | 0.50 | 0.49 |
|  | Transposed<16 MYA | 473 | 0.79 | 1.64 |
|  | proximal | 192 | 0.65 | 0.82 |
|  | tandem | 641 | 0.44 | 0.45 |
| 4 | all | 352 | 0.30 | 0.23 |
|  | alpha | 88 | 0.23 | 0.16 |
|  | beta | 85 | 0.24 | 0.21 |
|  | gamma | 22 | 0.31 | 0.29 |
|  | Transposed≥16 MYA | 78 | 0.40 | 0.29 |
|  | Transposed<16 MYA | 27 | 0.29 | 0.15 |
|  | proximal | 14 | 0.41 | 0.33 |
|  | tandem | 38 | 0.33 | 0.22 |
