# Supplementary material for: Expression Partitioning of Duplicate Genes at Single Cell Resolution in Arabidopsis Roots
Source: Front Genet. 2020 Nov 3;11:596150. doi: 10.3389/fgene.2020.596150 (PMC7670048; doi:10.3389/fgene.2020.596150)
Supplement: Supplementary Table S8 — Breakdown by expression class and duplication mechanism of 4,599 Wang et al. (2013) paralog pairs ubiquitously expressed in root cell clusters (RCC-u gene pairs). [file Table_8.docx]

**Supplemental Table 8.** Breakdown by expression class and duplication mechanism of 4,599 Wang et al., (2013) paralogue pairs ubiquitously expressed in root cell clusters (RCC-u gene pairs).

| Duplication type | Class | Count | RCC-u mixed | RCC-u both |
| --- | --- | --- | --- | --- |
| alpha | 0 | 0 | 0 | 0 |
|  | 1 | 94 | 94 | 0 |
|  | 2 | 590 | 148 | 442 |
|  | 3 | 970 | 690 | 280 |
|  | 4 | 31 | 17 | 14 |
| beta | 0 | 0 | 0 | 0 |
|  | 1 | 60 | 60 | 0 |
|  | 2 | 157 | 43 | 114 |
|  | 3 | 494 | 371 | 123 |
|  | 4 | 44 | 29 | 15 |
| gamma | 0 | 0 | 0 | 0 |
|  | 1 | 24 | 24 | 0 |
|  | 2 | 47 | 8 | 39 |
|  | 3 | 165 | 131 | 34 |
|  | 4 | 8 | 5 | 3 |
| Transposed≥16 MYA | 0 | 0 | 0 | 0 |
|  | 1 | 103 | 103 | 0 |
|  | 2 | 207 | 66 | 141 |
|  | 3 | 528 | 406 | 122 |
|  | 4 | 32 | 26 | 6 |
| Transposed<16MYA | 0 | 0 | 0 | 0 |
|  | 1 | 140 | 140 | 0 |
|  | 2 | 85 | 34 | 51 |
|  | 3 | 249 | 212 | 37 |
|  | 4 | 11 | 4 | 7 |
| proximal | 0 | 0 | 0 | 0 |
|  | 1 | 34 | 34 | 0 |
|  | 2 | 17 | 3 | 14 |
|  | 3 | 95 | 83 | 12 |
|  | 4 | 4 | 3 | 1 |
| tandem | 0 | 0 | 0 | 0 |
|  | 1 | 50 | 50 | 0 |
|  | 2 | 71 | 25 | 46 |
|  | 3 | 279 | 233 | 46 |
|  | 4 | 10 | 5 | 5 |
